# The Cattle Fever Tick, *Rhipicephalus microplus*, as a Model for Forward Pharmacology to Elucidate Kinin GPCR Function in the Acari

**DOI:** 10.3389/fphys.2019.01008

**Published:** 2019-08-07

**Authors:** Caixing Xiong, Dwight Baker, Patricia V. Pietrantonio

**Affiliations:** ^1^Department of Entomology, Texas A&M University, College Station, TX, United States; ^2^Department of Biochemistry and Biophysics, Texas A&M University, College Station, TX, United States

**Keywords:** leucokinin receptor, endogenous tick kinins, neuropeptide GPCR, dual-addition assay, small molecule screen, neurokinin antagonists, southern cattle tick

## Abstract

The success of the acaricide amitraz, a ligand of the tick tyramine/octopamine receptor (a G protein-coupled receptor; GPCR), stimulated interest on arthropod-specific GPCRs as targets to control tick populations. This search advances tick physiology because little is known about the pharmacology of tick GPCRs, their endogenous ligands or their physiological functions. Here we explored the tick kinin receptor, a neuropeptide GPCR, and its ligands. Kinins are pleiotropic insect neuropeptides but their function in ticks is unknown. The endogenous tick kinins are unknown and their cDNAs have not been cloned in any species. In contrast, more than 271 insect kinin sequences are available in the DINeR database. To fill this gap, we cloned the kinin cDNA from the cattle fever tick, *Rhipicephalus microplus*, which encodes 17 predicted kinins, and verified the kinin gene structure. We predicted the kinin precursor sequences from additional seven tick species, including *Ixodes scapularis*. All species showed an expansion of kinin paracopies. The “kinin core” (minimal active sequence) of tick kinins FX_1_X_2_WGamide is similar to those in insects. Pro was predominant at the X_2_ position in tick kinins. Toward accelerating the discovery of kinin function in ticks we searched for novel synthetic receptor ligands. We developed a dual-addition assay for functional screens of small molecules and/or peptidomimetics that uses a fluorescent calcium reporter. A commercial library of fourteen small molecules antagonists of mammalian neurokinin (NK) receptors was screened using this endpoint assay. One acted as full antagonist (TKSM02) with inhibitory concentration fifty (IC_50_) of ∼45 μM, and three were partial antagonists. A subsequent calcium bioluminescence assay tested these four antagonists through kinetic curves and confirmed TKSM02 as full antagonist and one as partial antagonist (TKSM14). Antagonists of NK receptors displayed selectivity (>10,000-fold) on the tick kinin receptor. Three peptidomimetic ligands of the mammalian NK receptors (hemokinin 1, antagonist G, and spantide I) were tested in the bioluminescence assay but none were active. Forward approaches may accelerate discovery of kinin ligands, either as reagents for tick physiological research or as lead molecules for acaricide development, and they demonstrate that selectivity is achievable between mammalian and tick neuropeptide systems.

## Introduction

**FIGURE 1:**
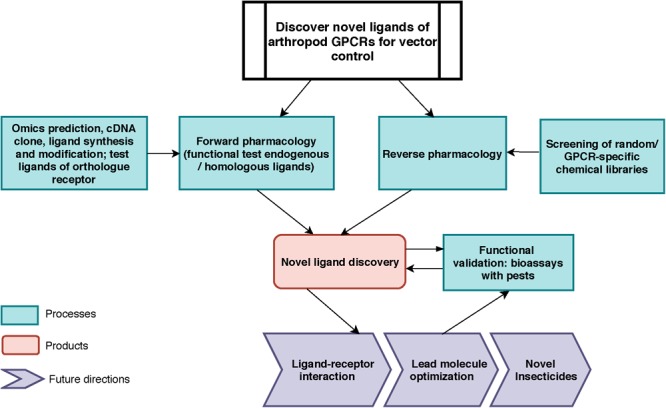
Toward vector control: General work-flow for the discovery of novel ligands for arthropod vector GPCRs. For forward pharmacological characterization of GPCR, endogenous ligands can be identified by *in silico* predictions, cloning, tissue extraction and purification, and subsequently be synthesized (e.g., neuropeptides). Ligands from known orthologs or pseudo-orthologous GPCRs from other animals could be tested (this work). For reverse pharmacology, GPCR-specific or random molecule libraries can be screened on the target GPCR. In both approaches, the active endogenous ligands and screened molecules will be validated through bioassays for their physiological functions *in vivo*. The outputs of this pipeline are: (1) endogenous ligand(s) identification; (2) novel small molecule discovery (3) knowledge of novel biological function of neuropeptide receptor; (4) data for receptor-ligand interactions modeling and for novel chemistry design.

The cattle fever tick or southern cattle tick, *Rhipicephalus microplus* (Canestrini), and the diseases it transmits cause significant losses to the livestock industry in tropical and subtropical regions of the world (Pérez de León et al., 2012). Considering the lack of effective vaccines against many of these vector-borne pathogens, vector control is still the most efficient approach to block disease transmission. However, worldwide distribution of tick resistance to the most commonly used acaricides, such as amitraz (formamidines), pyrethroids, organophosphates, and ivermectin was detected in tick populations (Guerrero et al., 2012; Pohl et al., 2012). In the near future, the current available pesticides will fail to control populations of these ticks as many exhibit multiple mechanisms of resistance with apparently no fitness cost. Pesticides safe to non-target species with novel modes of actions in vectors are needed. Here we describe a model study using a forward pharmacological approach to investigate a tick neuropeptide G protein-coupled receptor (GPCR) as potential target for tick control (Figure 1). This receptor, known as leucokinin-like peptide receptor (LKR) (accession AF228521), or myokinin receptor (Holmes et al., 2000, 2003) has been suggested as a promising novel target for pest control (Lees et al., 2010; Audsley and Down, 2015; Guerrero et al., 2016; Pietrantonio et al., 2018). A kinin peptidomimetic is antifeedant and lethal to the pea aphid (Smagghe et al., 2010), prevents the blood feeding to repletion in the kissing bug, *Rhodnius prolixus*, decreasing the chance of a successful molt (Lange et al., 2016) and triggers avoidance behavior in the mosquito *Aedes aegypti* when given in a sucrose solution (Kwon et al., 2016).

Kinin receptors are invertebrate-specific neuropeptide GPCRs (Pietrantonio et al., 2018). The kinin system is widely distributed in the Acari and in nearly every order of insects, except Coleoptera (beetles) (Halberg et al., 2015; Derst et al., 2016). Insect kinins are involved in many important physiological processes: they regulate diuresis (Hayes et al., 1989; Kersch and Pietrantonio, 2011), feeding (Al-Anzi et al., 2010; Kwon et al., 2016; Zandawala et al., 2018), pre-ecdysis (Kim et al., 2006) as well as tracheal air clearance post-ecdysis (Adams et al., 2000). In the fruit fly *D. melanogaster*, both leucokinin- and leucokinin receptor (LKR; also known as drosokinin receptor) loss-of-function mutant strains showed significant increases in resistance to desiccation, ionic stress (only tested in LK-mutant fly), and starvation (Cannell et al., 2016; Zandawala et al., 2018). Prior work has deorphanized kinin receptors of two vector species, the yellow fever mosquito *Ae. aegypti* (Pietrantonio et al., 2005) and the cattle fever tick *R. microplus* (Holmes et al., 2003), and found the kinin system is important in regulating diuresis and sugar feeding in *Ae. aegypti* (Kersch and Pietrantonio, 2011; Kwon et al., 2016). The most recent RNAi-mediated silencing of the kinin receptor in the cattle fever tick caused a reproductive fitness cost (Brock et al., 2019). While our cloning of this receptor represented the first known neuropeptide GPCR in the Acari, there has been no urgent need in establishing the endogenous ligand of the *R. microplus* tick. This was because receptor functional studies were performed using insect kinin core peptide analogs that activated the tick receptor. The endogenous ligands of the kinin receptor(s) feature a short C-terminal pentapeptide (Phe-X_1_-X_2_-Trp-Gly-NH_2_) as the minimal peptide core required for activity (Nachman et al., 2002). Insect kinins are among the most well characterized neuropeptides with currently 271 endogenous kinins identified in insects (Yeoh et al., 2017). In the synganglion of the tick *Ixodes ricinus*, we previously identified kinin peptide (leucokinin-like) immunoreactivity, and mass spectral analyses of synganglia of adult *R. microplus* and *Ix. ricinus* detected a strong signal at ∼1,008 Da, consistent with the mass of kinin peptides (Neupert et al., 2005). However, the endogenous cDNAs for kinin peptides have not yet been cloned in any tick species and tick sequences are unknown. Herein, we predicted and cloned the putative kinin cDNA which shows an amplification in the number of kinin ligands in *R. microplus*, similar to what was predicted for *Ix. scapularis* (Gulia-Nuss et al., 2016).

Previous functional studies with mosquito- and tick-kinin recombinant receptors tested insect kinin peptides, or kinin peptidomimetics designed for increased stability and/or penetration through the arthropod cuticle (Taneja-Bageshwar et al., 2006; Xiong et al., 2019). Results showed that the tick kinin receptor was a more permissive receptor; i.e., it was activated by more ligands and with lower EC_50_ than the mosquito receptor. There are no true orthologous mammalian receptors of the tick kinin receptor, which makes it an attractive potential selective target. However, the most similar mammalian receptors are the neurokinin receptors that mediate the biological actions of tachykinins (Pennefather et al., 2004). As also the tick kinin receptor is activated by the tachykinin of the stable fly (Holmes et al., 2003), we hypothesized that ligands of the mammalian neurokinin receptors could be active on the permissive tick kinin receptor.

To test this, a dual-addition assay was developed to determine the activity of peptidomimetics and small molecule ligands of neurokinin receptors on the tick kinin receptor. This assay allows discriminating agonist and antagonist activity in a single assay. Here we report the first small molecule ligands showing antagonistic activities on the tick kinin receptor. Although these small molecules did not exhibit high potency, this exploratory screen provides the methodological foundation for future screens of small molecule libraries in high-throughput mode. The results suggested mammalian NK receptor ligands displayed high selectivity over the arthropod kinin receptor. Additionally, the quantified activities of antagonists provide structure-activity data that helps define ligand-receptor interactions in computational models.

## Materials and Methods

### *In silico* Prediction of the Kinin Precursor cDNA Sequence in *R. microplus*

In search of the gene encoding the kinin precursor of the cattle fever tick, *R. microplus*, we first manually curated the protein sequence of the identified orthologous gene from the black legged tick, *Ixodes scapularis*, present in the genomic scaffold (DS680282| 583-1410) (Gulia-Nuss et al., 2016). This predicted nucleotide sequence of the kinin gene was translated in the six potential frames. Once the correct open reading frame encoding 19 putative kinin peptides was identified, the putative start codon was located but the stop codon could not be predicted within that scaffold. The curated protein sequence was used as the query for local TBLASTN analyses at the National Center for Biotechnology Information (NCBI)^1^ against the *R. microplus* (taxid: 6941) whole genome shotgun contig (WGS) (Rmi2.0; 2017), and its transcriptome shotgun assembly (TSA). The respective identified genomic fragments and transcripts were aligned with MegAlign (Lasergene, Madison, WI, United States) to assist with primer design for cDNA cloning. All sequence analyses in this study were performed with DNASTAR (Lasergene).

### Cloning the Kinin Precursor cDNA

The synganglia of females fed for 5 days (non-repleted) of the pesticide-susceptible Gonzalez strain of *R.* (*Boophilus*) *microplus* were used for cDNA synthesis. Details on tick dissection, mRNA extraction and 3′- and 5′-RACE ready cDNA syntheses were as described previously (Yang et al., 2013). To obtain the cDNA that encodes the putative kinin precursor from *R. microplus*, specific primers (Supplementary Table S1) were designed based on the predicted transcript available in NCBI (GEEZ01003316). For 5′- or 3′-RACE (Supplementary Figure S1), reactions were carried out in 50 μl volume, as follows: 2 μl of 5′- or 3′- RACE-ready cDNA of synganglia was added into the mix of 1 × Phusion HF buffer, 0.2 mM each dNTPs, 0.5 μM of UPM (5′, 3′ Race kit, Clontech^®^, Mountain View, CA), and 0.4 μM of RmkininP1R (for 5′ RACE) or RmkininP1F (for 3′ RACE), and 0.5 μl Phusion^®^ High-fidelity DNA polymerase (New England Biolabs^®^ Inc., Ipswich, MA). For 5′-RACE, touchdown PCR was as follows: 98°C for 30 s; followed by 5 cycles of 98°C for 10 s, 72°C for 1 min, followed by 5 cycles of 98°C for 10 s, 70°C for 30, 72°C for 1 min, followed by 25 cycles of 98°C for 10 s, 68°C for 30 s, 72°C for 1 min, with a final extension step of 72°C for 10 min. For 3′ RACE, touchdown PCR was as follows: 5 cycles of 98°C for 30 s, 72°C for 3 min, followed by 5 cycles of 98°C for 30 s, 70°C for 30 s, 72°C for 3 min, followed by 40 cycles of 98°C for 30 s, 68°C for 30 s, 72°C for 3 min, with a final extension step of 72°C for 5 min. The products from 5′- and 3′-RACE were purified with Zymoclean gel DNA recovery kit (Zymo^TM^ Research) and cloned into pCR^TM^2.1 Vector (Invitrogen). Chemical transformation was used to incorporate the plasmids containing PCR products into premade competent *E. coli* cells DH5α (Zymo^TM^ 5α) (Zymo^TM^ Research, Irvine, CA, United States). Transformants were screened by blue/white colony selection and 100 μg/ml ampicillin (Cayman Chemical, Ann Arbor, MI, United States). Plasmids were isolated using the Zyppy^TM^ plasmid miniprep (Zymo^TM^ Research) and sent to (Eton Bioscience Inc., San Diego, CA, United States) for Sanger sequencing. The full length of cDNA sequence was deduced by aligning the overlapping 5′- and 3′-end DNA fragments (Supplementary Figure S1A). To obtain the coding sequence of the cDNA (ORF) in a single product, gene-specific primers were designed (Rmk-ORF-F/R) outside of the ORF region to amplify the cDNA, using similar reagents as above (Supplementary Table S1). Specifically, in a 50 μl volume, 2 μl of 3′-ready cDNA was added into the mixture of 1 × Phusion HF buffer, 0.2 mM each dNTPs, 0.4 μM of both Rmk-ORF forward and reverse primers, and 0.5 μl Phusion^®^ High-fidelity DNA polymerase. The reaction was run at the following conditions: 98°C for 30 s, followed by 30 cycles of 98°C for 10 s, 72°C for 90 s, with a final extension step of 72°C for 10 min. The PCR product was purified, cloned into pCR^TM^2.1 Vector, and verified by sequencing as described above.

### *R. microplus* Kinin Gene Structural Characterization

The blastn “hits” obtained on the *R. microplus* genome with the cloned kinin cDNA sequence only overlapped the genome sequence toward the 3′ end of the cDNA. Thus, to define the structure of the putative kinin gene, i.e., to determine the precise number and length (bp) of introns and exons, several PCR reactions were performed (Supplementary Figure S1B). The genomic DNA template was extracted from the acaricide-susceptible Deutch strain of *R. microplus* because the Gonzalez strain used for cDNA synthesis is no longer available. Genomic DNA was extracted from one female tick using Zymo^TM^ *Quick*-DNA miniprep kit (Zymo^TM^ Research). To obtain the sequence of the predicted ORF, first, gene-specific primers located toward the 5′- and 3′- ends of the cDNA sequence (Rmk-ORF-F/R) (Supplementary Figure S1B) were used to amplify the genomic DNA. In a 50 μl volume, 100 ng of genomic DNA was added into the mixture of 1 × Phusion HF buffer, 0.2 mM of each dNTPs, 0.4 μM of both Rmk-ORF forward and reverse primers, and 0.5 μl Phusion High-fidelity DNA polymerase. The reaction was run at 98°C for 30 s, followed by 30 cycles of 98°C for 10 s, 72°C for 120 s, with a final extension step of 72°C for 10 min. A 4 kb PCR product was amplified, and it was purified with Select-a-Size DNA clean and concentrator^TM^ kit (Zymo^TM^ research, Irvine, CA, United States). Three forward primers and two reverse primers were designed to “walk” the ∼4 kb PCR product obtained for Sanger sequencing (Supplementary Figure S1B). Secondly, a gene-specific forward primer (Bmkinin-g-long-F) and a reverse primer outside the 4 kb product region (Bmkinin-g-long-R) were designed to amplify a 1,375 bp product that includes 40 bp toward the 3′ end of the kinin gene. For the reverse primer design, the sequence of the genomic contig (LYUQ01126194.1) was used (Supplementary Figure S1B). Thirdly, gene specific primers were designed based on the cDNA sequence (Supplementary Figure S1B) to amplify a ∼3.5 kb product containing additional 20 bp toward the 5′ end of the gene.

### *R. microplus* Kinin Peptide Precursor Characterization

The signal peptide of the translated *R. microplus* kinin peptide precursor sequence was predicted with SignaIP v. 5.0^2^ (Armenteros et al., 2019). The cleavage sites on the precursor were predicted following the principles summarized by Veenstra (2000). The conserved motif logos of the C-terminal pentapeptide of kinins in *R. microplus* and *Ix. scapularis* were created separately by WebLogo^3^ (Crooks et al., 2004).

### Prediction of Tick Kinin Peptide Precursors and Phylogenetic Analysis

The nucleotide sequences encoding the orthologous kinin precursors from other Acari species were predicted through tblastn on NCBI using the cloned kinin precursor of the *R. microplus* tick as query against the transcriptome data of Acari (taxi: 6933). The protein sequence was manually curated by translating the DNA sequence on ExPASy^4^. For species with more than one hit in the BLAST results, nucleotide sequences were downloaded and aligned by SeqMan Pro (DNASTAR Lasergene, Madison, WI, United States), before being used for protein curation. For specific genes for which the nucleotide sequences of two hits did not overlap, the encoding polypeptides were curated separately, and later combined from N-terminus to C-terminus retaining a gap between two polypeptides. This procedure applies for the predicted kinin precursor of *Amblyomma sculptum* and *Dermacentor variabilis*. To predict the *Ix. scapularis* kinin precursor, the transcript sequences identified by tblastn were used in combination with the sequence of the genome scaffold (DS680282) which had been previously reported to encode the kinin gene with 19 kinin peptides, with no other details provided (Gulia-Nuss et al., 2016). All the curated kinin precursor protein sequences start with methionine, and their predicted coding sequences end with a stop codon, except for that of *A. sculptum*, in which a stop codon was not present. To help verify the predicted methionine, all deduced tick kinin precursors were aligned using the Clustal W method with MegAlign (Lasergene). The kinin precursor sequence of the common bed bug, *C. lectularius*, was obtained from Predel et al. (2017). Additional insect kinin sequences were obtained from the DINeR database and references therein^5^ (Yeoh et al., 2017). Those sequences are from one hemipteran, *R. prolixus* [DAA34788.1] (Bhatt et al., 2014), and four dipterans, *Aedes aegypti* [AAC47656.1] (Veenstra et al., 1997), *Culex quinquefasciatus* [EDS35029.1] (Schooley et al., 2012), *Stomoxys calcitrans* [XP_013117801.1], and *Drosophila melanogaster* [NP_524893.2]. Fourteen protein sequences of putative arthropod kinin precursors were included in the phylogenic analysis. The protein sequences were first aligned by MAFFT with the iterative refinement algorithm E-INS-i, because of the occurrence of known insertions and deletions during the evolution of neuropeptide genes^6^, (Katoh and Standley, 2013), with the default online settings. The aligned sequences were processed through Mesquite version 3.6 (build 917) (Maddison, 2005) to convert the terminal gaps into missing data. Phylogeny was constructed using MrBayes version 3.2.6 (Ronquist et al., 2012) executable for Windows 64-bit with four chains and four runs in the mixed amino acid model for 1,000,000 generations. The traces of parameters were visualized in Tracer version 1.7.1 (Rambaut et al., 2018) to confirm that the four runs reached convergence. The consensus tree was generated with 10% burnins and output through FigTree version 1.4.4 (Rambaut, 2012).

### Cell Lines

BMLK3 is a CHO-K1 cell line stably expressing the southern cattle tick (*R. microplus*) kinin receptor, and served to characterize the functional activity of compounds on the receptor. Receptor cloning, transfection and selection of single clonal cell lines expressing this kinin receptor was reported previously (Holmes et al., 2000, 2003). A cell line similarly transfected with empty vector plasmid pcDNA3.1 (Invitrogen, Carlsbad, CA, United States) was designated as a “vector only” cell line and used as the negative control in all experiments. Cells were maintained in T-25 or T-75 flasks (CELLSTAR^®^, Greiner^®^ Bio-one) with maintenance medium consisting of F-12K medium (Corning^TM^ Cellgro^TM^, Mediatech, Inc., Manassas, VA, United States), fetal bovine serum (FBS) (10%) (Equitech-Bio, Kerrville, TX, United States) and 400 μg/ml G418 Sulfate (Gibco^®^ Invitrogen, New York, United States). Cells were maintained in a humidified incubator at 37°C, 5% CO_2_. Cells were incubated under the above conditions unless specified otherwise.

Two different calcium reporters (Fluo-8 AM or aequorin/coelenterazine) were utilized in dual-addition assays, which are described in detail in the sections below. Both assays allow the discrimination of agonistic or antagonistic activity of compounds. Briefly, a primary screen of compounds measured the endpoint fluorescence from cells cultured in a 384-well plate format. Compounds that showed potential activity in this screen were further tested at various concentrations with a kinetic assay that measured calcium bioluminescence in a 96-well plate format.

### Preparation of Small Molecule Library Plates

A commercial library of fourteen small molecule antagonists of mammalian neurokinin receptors (NK1-3) was purchased from Tocris^®^ Bioscience (R&D System, Bristol, United Kingdom) (for information on chemicals see Supplementary Table S2) to be screened on the BMLK3 cells in 384-well format. Additionally, thapsigargin, which discharges intracellular Ca^2+^ stores by inhibition of the Ca^2+^ ATPase in endoplasmic reticulum (Thastrup et al., 1990), and 2-APB (2-aminoethoxydiphenyl borate), which blocks of store-operated Ca^2+^ entry and may block InsP3-induced Ca^2+^ release (Bootman et al., 2002), were purchased from Tocris^®^ Bioscience and used as positive and negative controls, respectively, for the Ca^2+^ signal assay readout.

All small molecule stock solutions (for library and controls) were prepared in 100% DMSO (Sigma-Aldrich, St. Louis, MO, United States) and aliquoted and stored at -20°C before use. For the initial screening in the fluorescence assay, compounds were initially prepared in a V-bottom, 384-well plate (Corning^®^, NY, United States) in Hanks’ Saline Buffer containing 20 mM Hepes (HHBS), 2% DMSO, at a 10× concentration of the final concentration in the assay. The sixteen small molecules were serially diluted in this plate into 22 dosages using a dilution ratio of 1:1.4, starting at 1 mM (except thapsigargin started at 100 μM, Supplementary Table S3) using an automatic-8 channel EPMotion^TM^ liquid handler (Eppendorf Biotech company, Hamburg, Germany).

Ten selected molecules, which were either active in the first screening or could not be dissolved in 2% DMSO, were further prepared in a V-bottom 384-well plate in HHBS, 10% DMSO at a 10× concentration of the final concentration in the assay; thapsigargin and 2-APB were also prepared in the same solvent. The twelve small molecules (including thapsigargin and 2-APB) were serially diluted in the plate (dilution ratio 1:1.4) into 10 dosages starting from various concentrations (10 μM to 1 mM) depending on the solubility of each molecule (Supplementary Table S4, Panel A). In this “library subset” plate, each concentration of the small molecules was dispensed into a duplicate well for testing both the kinin-receptor expressing cell line (BMLK3 cells) and the cells transfected with the vector plasmid only, respectively. In both 384-well library plates, 64 wells in four edge columns were filled with blank solvent (first addition) (Supplementary Tables S3, S4).

### Preliminary Screen of a Small Molecule Library in an End-Point Fluorescence Assay

The screening of the potential antagonists on BMLK3 cells was performed with an endpoint fluorescence assay in a black/clear 384-well plate (CELLSTAR^®^, Greiner Bio-one, 781077) coated with Poly-D-Lysine (Sigma-Aldrich). This assay uses Fluo-8 AM (Fluo-8 Calcium Flux Assay Kit - No Wash, Abcam^®^, Cambridge, United Kingdom) as the calcium indicator. Unless specified, the pipetting steps in the 384-well plate fluorescence assay were performed by an automated CyBio^®^ Well Vario System using a 384 pipette-head that allows to simultaneously deliver a volume of up to 60 μl per well. The screening of the first library prepared in 2% DMSO was performed on BMLK3 cells only (Supplementary Table S3). The screening of the second library plate prepared in 10% DMSO (“library subset”) was tested with half of plate with BMLK3 and another half with vector only cells (Supplementary Table S4).

BMLK3 or vector only cells were cultured in T-75 flasks. When they reached about 90% confluency, they were trypsinized and suspended in F-12K medium containing 1% FBS and 400 μg/ml of G418 Sulfate at 4 × 10^5^ cells/ml to be seeded in 384-well plates. For this, the cell suspension (25 μl; ∼10,000 cells/well) was dispensed into all 384 wells of the plate. To distribute cells evenly, mixing was by aspirating and immediately re-dispensing 10 μl of the applied cell suspension three times. Plates were incubated overnight at 37°C under 5% CO_2_. On the next day, cells were prepared for the assay following the kit’s instructions: the old media in the assay plate was fully removed by inverting the plate and gently blotting it on paper towels, and media was replaced with 45 μl of Fluo-8 AM loading dye (1×). The plate was incubated at 37°C under 5% CO_2_ for 30 min, then equilibrated at room temperature in the dark for 30 min. The screening of the small molecule library was achieved by a dual-addition assay. The “first addition” consisted of 5 μl of either the blank solvent or a 10 × solution of the potential antagonist transferred from the 384-well library plate, to reach 1 × concentration in the wells. After 5 min incubation with cells, a second addition of 5.6 μl of 10 μM kinin receptor-specific agonist peptide (FFFSWGamide) resulted in a final concentration of 1 μM. The calcium fluorescence signal was read by a Varioskan LUX^TM^ (Thermos Scientific, Waltham, MA, United States) plate reader set for fluorescence plate-mode with Ex/Em = 490/525 nm and kept at 29°C. Endpoint responses were read immediately after the first addition and 5 min after the second addition of agonist. The plate was read from both forward and reverse orientations by rotating the plate (180°) on the instrument to compensate the variation in signal kinetics during the lapse in plate reading, because there was a 2 min lag time between the readings of the first and the last well. The response to each addition was represented as the average value of both forward and reverse plate readings (Supplementary Tables S3, S4, Panels B,C). The antagonist activity was calculated as the percentage of the response to 1 μM FFFSWGa of cells that had been incubated with the putative antagonist compound in comparison to the response of cells that had been incubated with blank solvent only. A compound was considered to have antagonistic activity, if it minimally inhibited 50% of the response to the agonist FFFSWGa (1 μM) applied in the second addition. These candidates were selected for further validation in a dose-response kinetic calcium mobilization bioluminescence assay.

### Kinetic, Dose-Response Calcium Mobilization Bioluminescence Assay

The calcium bioluminescence assay was used for the kinetic analysis of dose-responses to the compounds identified in the primary screen. Aequorin is the calcium reporter, transiently expressed in the BMLK3 cells (Lu et al., 2011b). This “dual-addition” kinetic assay was conceived to characterize the diverse activity patterns the putative ligands may display. The “first addition” consists of the compound being tested, either a small molecule or a peptidomimetic; the bioluminescence response elicited is measured for 30 s (if the compound is an agonist there will be bioluminescence response during this first 30 s period). Immediately after, at 32 s, a “second addition” with a single concentration of agonist follows (FFFSWGa, 1 μM), and the response continues to be measured for another 30 s. It is important to emphasize that the response measured after this addition of agonist is influenced by the presence of the unknown compound applied in the first addition. If the compound is an antagonist, the response to the agonist applied in the second addition will be reduced with respect to that of the positive control (buffer + agonist). The pharmacological activity of the unknown molecule can be determined based on the integrated area under the bioluminescence response curve registered during both these 30 s periods (total bioluminescence expressed as average bioluminescence units per second). That is, it can be inferred if the unknown molecule is a full agonist, full antagonist, partial agonist, partial antagonist, or an allosteric modulator. This approach is widely applied in GPCR drug discovery (Ma et al., 2017).

Selected small molecules were solubilized in 1 × DMEM medium (Gibco^®^, Invitrogen) with 10% DMSO and prepared from 500 nM to 500 μM as 5× of the final concentration. In addition, three peptidomimetic ligands of neurokinin receptors were purchased from Tocris^®^ Bioscience: one agonist, hemokinin 1 (human), and two antagonists, antagonist G and spantide I. These peptidomimetics were solubilized in 80% acetonitrile: 0.01% trifluoroacetic acid and then aliquoted (100 nm per tube) and freeze-dried; the dry peptidomimetics were stored at -20°C before use. For the assay, the peptidomimetics were solubilized and diluted in assay buffer (1 × DMEM) containing 1% DMSO from 10 nM to 100 μM as 10× of the final concentrations in the assay. All compounds, either small molecules or peptidomimetics were tested in three independent replicates, each with 2-3 wells as pseudo-replicates. Responses from each assay were calculated as the average of individual responses from the pseudo-replicate wells.

The cells preparation was described in detail elsewhere (Lu et al., 2011b). In brief, the BMLK3 and vector-only cells were cultured to ∼ 90% confluency in T-25 flasks. The cells were trypsinized and suspended in maintenance medium, counted, and diluted to 1 × 10^5^ cells/ml; 2 ml of this cell suspension was placed into each well of 6-well-plates (CELLSTAR^®^, Greiner Bio-one). After overnight incubation, when the cells reached a confluency of 40–60%, old medium was replaced with 1 ml of serum-reduced Opti-MEM^TM^ medium (Gibco^®^, Invitrogen) in each well. Following the instructions of the transfection reagent manufacturer, cells in each well of the 6-well plate were transiently transfected with 1 μg mtAequorin/pcDNA1 plasmid mixed in 4 μl of FuGENE6 (Promega, Madison, WI, United States) and 96 μl of Opti-MEM^TM^ medium. After 6 h of incubation, the old medium was replaced with 2 ml of F-12K medium with FBS (10%) (antibiotic-free medium). Following 24 h incubation, cells were trypsinized, seeded into white/clear 96-well-plates (CELLSTAR^®^, Greiner Bio-one) (20,000 cells/well in 100 μl of antibiotic-free medium), and incubated overnight until they reached optimal confluence of 80%. BMLK3 cells and vector only cells were prepared in the same plate to test different concentrations of each compound. To reconstitute the aequorin-complex, cells were incubated with 90 μl per well of calcium-free DMEM (1×) containing coelenterazine (5 μM) (Regis^TM^ Technology, Inc., Morton Grove, IL, United States). After 3 h of incubation in the dark, cells were ready for the assay. The “dual-addition” assays were performed with a Clariostar^TM^ (BMG LABTECH, Chicago, IL, United States) plate reader set at 29°C and for bioluminescence and “well” mode at 469 nm emission wavelength. The two additions were performed by the built-in injectors of the Clariostar^TM^plate reader. The first addition was performed after 2 s of initiating the recording of the bioluminescence response, which continued for 30 s. This addition applied either 22.5 μl (5×) of certain small molecule antagonist at a specific concentration or blank solvent, or 10 μl of peptidomimetic (10×). At 32 s, a second addition of 12.5 μl of agonist peptide FFFSWGa was executed with the second injector into the same well containing the test compound or blank solvent, to reach a final concentration of 1 μM. For peptidomimetics, 11 μl were applied. Recording continued for another 30 s; in sum, the kinetic response of each well was recorded for a total of 65 s at 1 s intervals. The same procedure was performed for each concentration of the ligand tested. The kinetic response to both additions were recorded immediately after addition for 30 s, with 1 s interval, and is expressed as bioluminescence units (BU) per second. The total response of the cells to each addition was represented as the percentage of the averaged BU per second obtained in each of the two 30 s-ranges, relative to the average BU to the PC (blank solvent + 1 μM FFFSWGa) in the 2nd range (average BU of the PC curve from 35 to 65 s).

### Effect of a Prolonged Pre-incubation With Various Concentrations of TKSM14 on the Agonist-Induced Response

Because TKSM14 displayed antagonistic activity in the fluorescence assay but not in the calcium bioluminescence assay, a modified calcium bioluminescence assay was performed in which the incubation time (previously 30 s) was increased to 5 min, as in the fluorescence assay. The first addition was performed manually to add 22.5 μl of the blank buffer or different dosages of the TKSM14 (5 × solutions), to achieve final concentrations of 1, 10, 30, 50, and 100 μM. The second addition was performed with the built-in injector of the Clariostar^TM^ plate reader (12.5 μl of the 10× agonist FFFSWGa solution for a final concentration of 1 μM), as described in the previous section. The response to the agonist in the second addition was recoded for 30 s, at 1 s intervals. Similarly, as before, three independent assays were performed, with each assay containing 3–4 pseudo-replicates. Responses from each assay were calculated as the average of individual responses from the pseudo-replicate wells. The antagonistic activity was represented as a percentage of the average bioluminescence units obtained during the 30 s after the second addition (wells containing both the small molecule being analyzed and FFFSWGa), divided by the average bioluminescence units obtained from wells containing FFFSWGa only.

### Statistical Analyses

All statistical analyses were performed with Prism 6.0 (GraphPad Software, La Jolla, CA, United States). In the end-point fluorescence assay, dose-response curves and IC_50_ values were calculated with “log [inhibitor] vs. response – Variable slope (four parameters)” function in Prism 6.0. IC_50_s of the antagonists were defined as the concentration of antagonist that inhibits agonist response in the mid-range of the respective fitted dose-response curve (this is not the concentration that inhibits 50% of the agonist response). In the kinetic calcium bioluminescence assay, to determine the statistical significance of the inhibitory effect from various concentrations of the same compound, the response after the second addition for each concentration within each molecule was compared to the corresponding blank control by one-way ANOVA (*n* = 3) followed by Tukey’s multiple comparisons test.

## Results

Our long-term approach for discovering novel ligands for arthropod GPCRs is summarized in Figure 1. In this study, we focused on processes on the left of the figure, by using transcriptomics and genomic data to clone the kinin cDNA, followed by forward pharmacological approaches toward the identification of novel ligands for the kinin receptor of the southern cattle tick, *R. microplus*.

### *R. microplus* cDNA Sequence and Gene Structure

We first identified the predicted 332 amino acid residue protein sequence of the putative kinin precursor of *Ix. scapularis* from a genome fragment (DS680282: 583-1410) (Gulia-Nuss et al., 2016). Although it was reported that the gene encoded 19 kinins (Gulia-Nuss et al., 2016), the exact sequences of kinins were not provided. Herein, we listed the sequence of the 19 paracopies of putative bioactive kinins from *Ix. scapularis* (Table 1). Using this curated precursor protein as query, the tBLASTn results against WGS assembly and TSA of *R. microplus* (taxid: 6941) identified a genomic DNA fragment of accession number LYUQ01126194.1 and four transcripts with high similarities. Our cloning of the putative kinin precursor cDNA from *R. microplus* verified the full-length cDNA sequence is 1,398 bp long encoding a 1,227 bp open reading frame (ORF). The predicted precursor protein of 409 amino acid residues has a predicted molecular mass of 44.72 kDa (GenBank QDO79406) (Supplementary Figure S2). The 5′- and 3′-untranslated regions (UTRs) are 85 bp and 86 bp, respectively.

**Table 1:** Predicted bioactive kinins from *Rhipicephalus microplus* and *Ixodes scapularis.*

Name	*Rhipicephalus microplus*	Predicted molecular weight (Daltons)
Rmkinin1	QFSPWGa	720
Rmkinin2	LHPVDIAVRAADLFSPWGa	1963
Rmkinin3	DKDQTFNPWGa	1206
Rmkinin4	AGDHF**G**SWGa	932
Rmkinin5	DTFSAWGa	782
Rmkinin6	QQDSKNAFSPWGa	1363
Rmkinin7	AVRSPTARNDAARAKQEDGEEDEERSF**A**PWGa	3446
Rmkinin8	GTGEDQAFSPWGa	1250
Rmkinin9	GDDGDTSF**T**PWGa	1253
Rmkinin10	DDRFNPWGa	**1005**
Rmkinin11	EGPFSPWGa	875
Rmkinin12	DGSNKEGFFNPWGa	1454
Rmkinin13	GADDPFNPWGa	**1074**
Rmkinin14	QDSFNPWGa	949
Rmkinin15	EDGVF**R**PWGa	**1061**
Rmkinin16	EDNVF**R**PWGa	1118
Rmkinin17	EGNVF**G**PWGa	961
	***Ixodes scapularis***	
Ixkinin1	QFSPWGa	720
Ixkinin2	GDKQPEDEAFNPWGa	1589
Ixkinin3	ENDKDKELSFNPWGa	1678
Ixkinin4	GSFSSWGa	726
Ixkinin5	DTF**G**SWGa	768
Ixkinin6	DTF**G**PWGa	768
Ixkinin7	DTF**G**PWGa	768
Ixkinin8	DTF**G**PWGa	768
Ixkinin9	DTF**G**PWGa	768
Ixkinin10	DTF**G**PWGa	768
Ixkinin11	QDKESGFNPWGa	1263
Ixkinin12	DPFNPWGa	831
Ixkinin13	EDKNAFSPWGa	1149
Ixkinin14	DQNFNPWGa	976
Ixkinin15	TTKDSTFSPWGa	1225
Ixkinin16	EGPFNPWGa	902
Ixkinin17	GDSDTAF**A**PWGa	1122
Ixkinin18	DNNFNPWGa	962
Ixkinin19	DNGNKDSSFSPWGa	1409

*Under name, ordinal numbers reflect the order in which each sequence appears from the 5′ to 3′ direction either in the cDNA (*R.m*.) or in the predicted gene (*Ix*.). The bolded predicted molecular weights were similar to previously predicted kinin masses (approximately 1,009 Da) (Neupert et al., 2005).*

**FIGURE 2:**
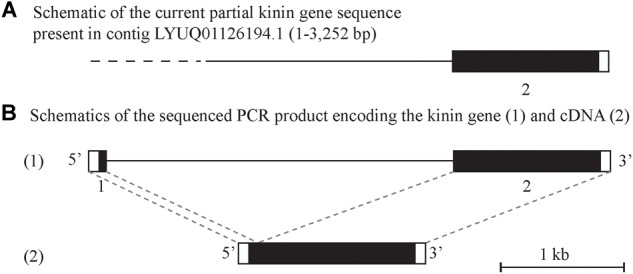
Schematic of the kinin gene and cDNA from *Rhipicephalus microplus*. The black boxes represent the protein-encoding regions and the white boxes are the untranslated regions on the 5′ and 3′-end of the kinin gene or cDNA. **(A)** Depicts a genome contig fragment (the full length of 7,784 bp is not represented here) encoding the 3′-end fragment of the predicted *R. microplus* kinin gene (position 1 to 3,252). The dashed line represents the sequence gap at the 5′-end of the kinin gene in the genome assembly (Rm2.0; NCBI). **(B)** (1) Depicts the amplified 4.1 kb PCR product sequenced that encodes the kinin gene. The gene is composed of two exons (140 and 1,254 bp, respectively, represented by boxes) and one intron (2.7 kb black line). This structure was verified by aligning the gene sequence with the 1398 bp cDNA (2) obtained in this study.

Alignment of the cloned cDNA to the genome sequence revealed that the 5′-end was missing in the genome, as determined by BLAST (Figure 2A). We were able to obtain this missing sequence by genomic PCR amplification using a primer designed based on the sequence of the cDNA 5′-end (Supplementary Figure S1 and Supplementary Table S1). By further amplification of the genomic DNA with gene-specific primers we sequenced the putative kinin gene of *R. microplus*, that is encoded in approximately 4.1 kb (GenBank MK875970) (Figure 2B), composed of two exons and one intron. The first exon is 140 bp long and the second exon is 1,253 bp long; in between there is an intron of ∼ 2,700 bp. Although the genomic DNA and cDNA sequences obtained were from different strains of *R. microplus*, the alignment of their ORF region showed 99% identity.

### Analysis of the *R. microplus* Kinin Precursor

**FIGURE 3:**
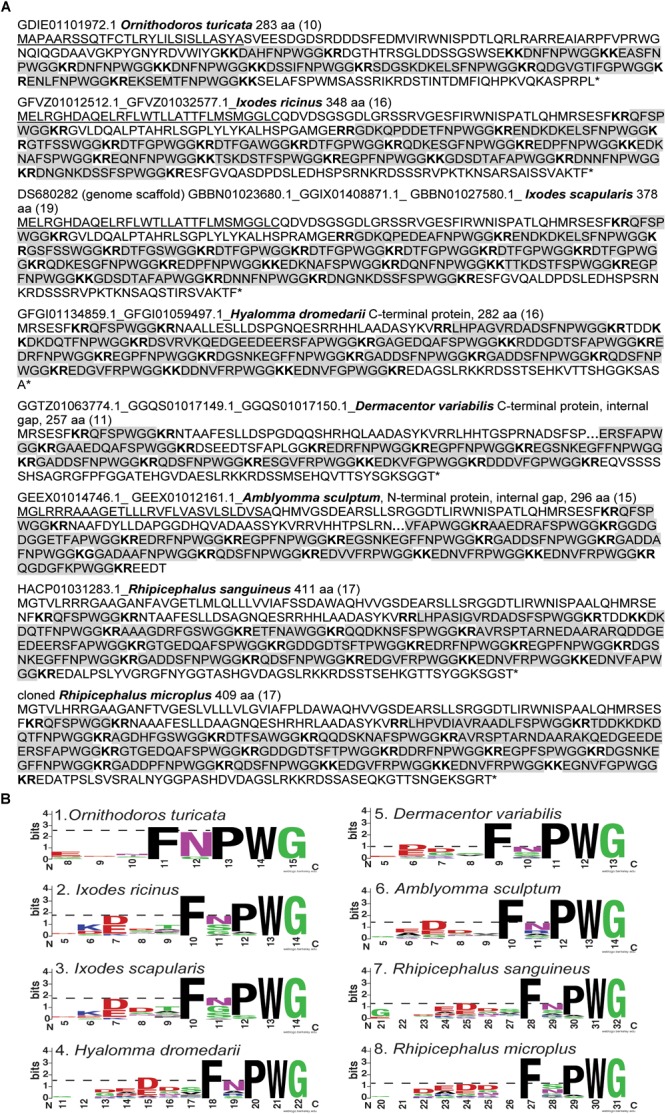
Amino acid sequences of kinin precursors from eight tick species. **(A)** Amino acid sequences were manually curated after mining the available transcriptomes on NCBI. The number of kinin paracopies in each precursor are denoted in the parentheses. The gaps within the protein are denoted with “**…**.” The signal peptides predicted by SignalP 5.0 were underlined. The predicted cleavage sites are in bold, and potential bioactive kinins containing the kinin C-terminal motif, FX_1_X_2_WG-amide, are highlighted with gray color. The sequence of kinin precursor from *Ixodes scapularis* was curated based on the putative kinin gene in the genome scaffold (DS680282), while the additional N- and C- terminal sequences were deduced from sequences of three transcripts (Supplementary Figure S3). The number 1 to the left of the *Amblyomma sculptum* sequence points to an unusual cleavage site (K) or a potential sequencing error at this site (K followed by G) (in black square frame). The version numbers are listed on the top of each sequence (accession numbers are identical except do not contain the 0.1 at the end). **(B)** Sequence logos of the kinins from eight tick species. The logos were created by WebLogo (Crooks et al., 2004) using the amino acid sequences from each tick kinin precursor between predicted cleavage sites (highlighted in panel **A**). The overall height of each letter stack indicates the sequence conservation at that position (measured in bits), whereas the height of amino acid symbols within the stack reflects the relative frequency of the corresponding residue at that position. On the *X* axis, the numbers refer to the amino acid position within the alignment, which was anchored to the conserved C-terminal amidated-glycine residue, similarly as shown in the DINeR database (Yeoh et al., 2017). Toward the N-terminus, logos begin at the position within the alignment where bits are above 0 value. Polar amino acids containing an amide group (Q, N) are in purple, other polar amino acids (G, S, T, Y, C) are in green, basic (K, R, H) are in blue, acidic (D, E) in red and hydrophobic (A, V, L, I, P, W, F, M) amino acids are in black. The dashed-line indicates the bit value of the amino acid residue at the first variable position (X_1_) of the kinin C-terminal pentapeptide motif (FX_1_X_2_WGamide).

The first 38 amino acid residues of the *R. microplus* kinin precursor were predicted with SignalP v 5.0 as belonging to a signal peptide with only 50% probability, denying such conclusive identification. Seventeen paracopies of putative bioactive kinins were encoded by this single cDNA (Table 1). Three of them (Rmkinin 10, 13, and 15) have a predicted molecular weight of about 1,009 Da, which was the mass of putative *R. microplus* kinin detected through mass spectrometry in a previous study (Neupert et al., 2005). The *R. microplus* kinins showed the canonical critical residues and necessary characteristics for activity of the insect kinins (FX_1_X_2_WGa): Phe and Trp at the first and fourth position of the pentapeptide minimally active core, respectively, as well as the amidated Gly at the fifth position. Noticeably, the second variable position (X_2_) was dominated by Pro; only two out of 17 kinin paracopies from *R. microplus* have a different amino acid other than Pro on the X_2_ position (Table 1). This consensus pattern of the kinin C-terminal pentapeptide (FX_1_PWGa) was prevalent in other tick species as well (Table 1 and Figure 3A).

### Kinin Precursors From Other Tick Species

Using the *R. microplus* kinin precursor as query for TBLASTN against the transcriptomes of all Acari (taxid: 6933) on NCBI, we predicted amino acid sequences of the putative kinin precursors from another seven tick species. Four full amino acid sequences were obtained, those from the relapsing fever tick, *Ornithodoros turicata*, the castor bean tick, *Ix. ricinus*, the brown dog tick, *R. sanguineus*, and the curated kinin precursor of *Ix. scapularis.*

The precursor from *Ix. scapularis* was predicted by aligning the kinin precursors deduced from three transcripts and the genomic scaffold. Missing sequences were identified from each of these: the transcripts provided a sequence of 23 amino acid residues at the N-terminus containing part of the signal peptide, and an additional C-terminal sequence of 23 amino acid residues followed by the stop codon. These sequences are absent in the genomic scaffold. However, the protein deduced from the transcriptome lack 40 amino acid residues, which are present in the genomic scaffold, distributed in two gaps within positions 250–293 in the alignment (see Supplementary Figure S3). These gaps may have arisen from errors in the transcriptome assembly because this genomic region encodes four repeats of the sequence “DTFGPWGGKR.” Therefore, the hypothetical kinin precursor from *Ix. scapularis*, is predicted as a protein of 378 amino acid residues (Figure 3A).

Three partial kinin precursors were predicted from the American dog tick, *Dermacentor variabilis*, and from *Amblyomma sculptum* and *Hyalomma dromedarii*. The former two were predicted with a gap retained in both amino acid sequences (Figure 3A). Whereas the *H. dromedarii* precursor, while missing approximately 70 amino acid residues at the N-terminus, should contain all the kinin paracopies as inferred from the alignment of tick kinin precursors (Supplementary Figure S3). Similarly, the deduced amino acid sequence of *D. variabilis* kinin precursor appeared to miss the same 70 residues sequence at the N-terminus (Supplementary Figure S3). Among the seven deduced protein sequences, only the *A. sculptum* kinin precursor lacked the stop codon. Among the eight tick kinin precursors analyzed, SignalP 5.0 predicted the signal peptide from those of *O. turicata, Ix. scapularis, Ix. ricinus*, and *A. sculptum* (Figure 3A). For *O. turicata*, the signal peptide includes the first 28 amino acid residues, with a cleavage site between residues A and S (Figure 3A). For the latter three species, the signal peptide includes the first 30 amino acid residues, with the cleavage site between C and Q for *Ixodes* spp., and between A and Q for *A. sculptum*.

Putative enzyme cutting sites, characterized by contiguous basic residues (Veenstra, 2000), and those for kinin sequences were predicted in the translated precursor sequences (Figure 3A). The number of kinin paracopies was high, especially in hard ticks. In the curated full-length kinin precursor of four hard tick species, 17 paracopies of kinins were predicted, in average. The transcript of the only soft tick kinin gene analyzed (*O. turicata)* is predicted to encode 10 kinin paracopies.

Within the functional C-terminal pentapeptide (FX_1_X_2_WGamide) core of insect kinins, the first (F), fourth (W), and fifth (G) amino acid residues are highly conserved, and similar conservation was observed in tick kinins. However, the second variable position predominantly featured proline in all eight tick species (Figure 3B), and the first variable position showed lower conservation in all analyzed tick species. Asparagine (N) predominately occupied the X_1_ position in the soft tick *O. turicata* kinins, while in the *Ixodes* ticks (Prostriata), the X_1_ position was equally occupied by either N, G, or S. The amino acid residue in the X_1_ position was even less conserved (lower bits value) in ticks of the Metastriata group (Figure 3B, 4–8).

**FIGURE 4:**
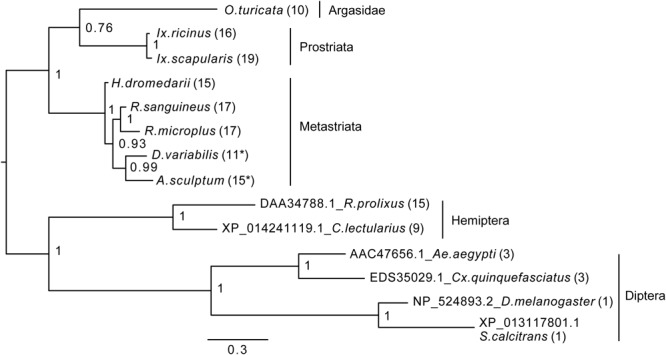
Bayesian phylogenetic analysis of kinin precursor. The phylogenetic tree was constructed in MrBayes with four chains and four runs of the mixed amino acid model for 1,000,000 generations with a 10% burnins. Node values are Bayesian posterior probability rounded to two significant figures. Scale refers to branch length and represents the probability of an amino acid substitution along that interval. The number of kinin paracopies encoded by each precursor is denoted in the parentheses. “^∗^” Indicates there could be more kinin paracopies present in this species as the predicted amino acid sequence likely only encodes a fragment of the kinin precursor due to gaps present in the predicted transcripts in NCBI. The curated protein sequences and version numbers of corresponding transcript sequences are in Figure 3A.

**FIGURE 5:**
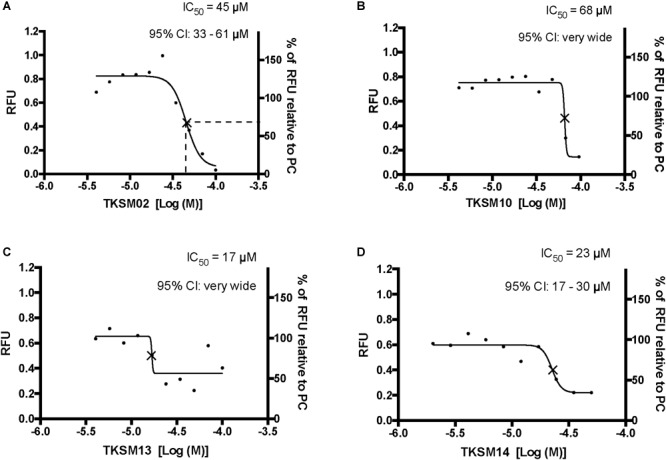
Four small molecules antagonized the tick kinin receptor activity. Only four molecules out of the 10 tested exhibited antagonistic activities. **(A–D)** Dose-responses of the tick kinin receptor (in the BMLK3 cell line) to small-molecule ligands of mammalian neurokinin receptors were measured in a calcium fluorescence assay. Ten dosages were applied for each compound, starting from 100 μM (except TKSM14, starting from 50 μM) and following a 1:1.4 dilution series. The antagonist activity of 10 selected small molecules was tested by first dispensing the blank buffer or small molecules at various concentration [Log (Molar) on the *X*-axis] from the library plate into the assay plate. This was followed by incubation with the cells for 5 min, after which the kinin agonist (1 μM FFFSWGa) of the tick kinin receptor was dispensed into all wells and incubated for another 5 min. The end-point relative fluorescence units (RFU) from each well was represented as the average RFU (left *Y*-axis) of two end-point reads obtained by inverting the orientation of the plate in a Varioskan LUX^TM^ (Thermos Scientific, Waltham, MA, United States) plate reader. Additionally, the right *Y*-axis represents the percentage of RFU relative to the averaged responses from cells to the PC (blank buffer + agonist). Non-linear dose-response curves IC_50_s were defined as the concentration of antagonist that inhibits agonist response in the mid-range of its respective fitted dose-response curve and they are marked with a cross (×) on the curve. The IC_50_s and corresponding 95% confidence intervals of IC_50S_ were calculated with “log(inhibitor) vs. response- Variable slope (four parameters)” in GraphPad 6.0. The IC_50s_ shown here are not the concentrations that inhibit 50% of the agonist response.

**FIGURE 6:**
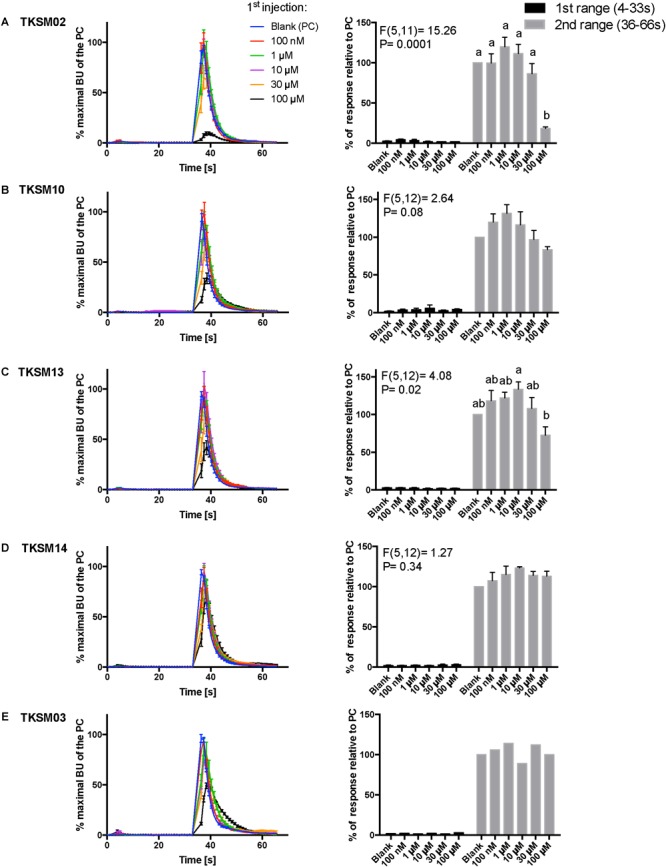
Kinetic dose-responses of the BMLK3 cell line to five selective small molecules. Kinetic bioluminescence responses of tick kinin receptor recombinant cells to **(A–D)** four antagonist candidates (*n* = 3), and **(E)** TKSM03 [negative control (*n* = 1)] in the “dual-addition” calcium bioluminescence assay. All small molecules were tested at five different concentrations from 100 nM to 100 μM. Two additions were performed by the two injectors built-in the Clariostar^TM^ plate reader (BMG LABTECH) as follows: the first addition of various concentrations of small molecules (5×) or corresponding blank buffer, was performed at 2 s after initiation of the recording. The response to the first addition was recorded for 30 s with 1 s intervals (first range). At 32 s, the second addition of the agonist of tick kinin receptor used in this study (1 μM FFFSWGa) was performed in the same well, and the cell response was recorded for another 30 s with 1 s intervals (second range). Curves on the left of each panel depict in different colors the kinetic response in bioluminescence units (BU) per second to each concentration (4–9 wells) from three independent assays (except TKSM03 *n* = 2 wells from one assay). The *Y*-axis represents the percentage BU of each concentration normalized to the maximal BU of the positive control (blank buffer + agonist, blue curve) (mean ± SEM). The histogram on the right of each panel shows sequentially the total BU responses of cells to the two additions, the first (black) and second (gray) range recordings, respectively. The total response in each range was expressed as the percentages of the average BU (per second) obtained in each of the two 30 s-ranges, relative to the average units to the PC in the second range (average BU of the blue curve at 35–65 s). In each independent assay, the total response to each concentration of each small molecule in the first addition or to the agonist in the second addition was calculated as the average of 2–3 pseudo-replicate wells (*Y* axis: mean ± SEM; *n* = 3 independent assays). None of the tested compounds showed agonist activity. Statistical differences in inhibitory activities between different concentrations of each compound were analyzed by one-way ANOVA (*n* = 3) followed by Tukey’s multiple comparison test (*p* ≤ 0.05) with GraphPad 6.0 (GraphPad Software, La Jolla, CA, United States). Bars with different letter superscripts indicate significant differences.

**FIGURE 7:**
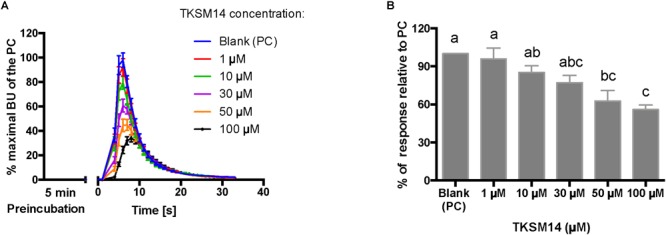
BMLK3 preincubation (5 min) with dosages of TKSM14 before agonist addition, enhances its antagonistic activity in the calcium bioluminescence assay. **(A)** The kinetic responses of BMLK3 cells to the agonist (1 μM FFFSWGa) after being incubated with various concentrations of TKSM14 (1–100 μM) or blank solvent for 5 min. The response was recorded for 30 s at 1 s intervals by the Clariostar^TM^ plate reader (BMG LABTECH). The kinetic response is represented as the percentage of the maximal bioluminescence units (BU) of the positive control (PC = blank buffer + agonist, blue curve) in each assay (Mean ± SEM, *n* = 9–10 wells from three independent assays). **(B)** The total cellular response in each well is expressed as the percentage of the average BU (per second) obtained in the 30 s relative to the average response units to the PC in 30 s. Three independent assays were performed. Responses from each assay were calculated as the average of individual responses from 3-4 pseudo-replicate wells (*Y* axis: mean ± SEM). Statistical differences in inhibitory activities between different treatment were analyzed by one-way ANOVA (*n* = 3) followed by Tukey’s multiple comparison test (*p* ≤ 0.05) with GraphPad 6.0 (GraphPad Software, La Jolla, CA, United States). Bars with different letter superscripts indicate significant differences.

**FIGURE 8:**
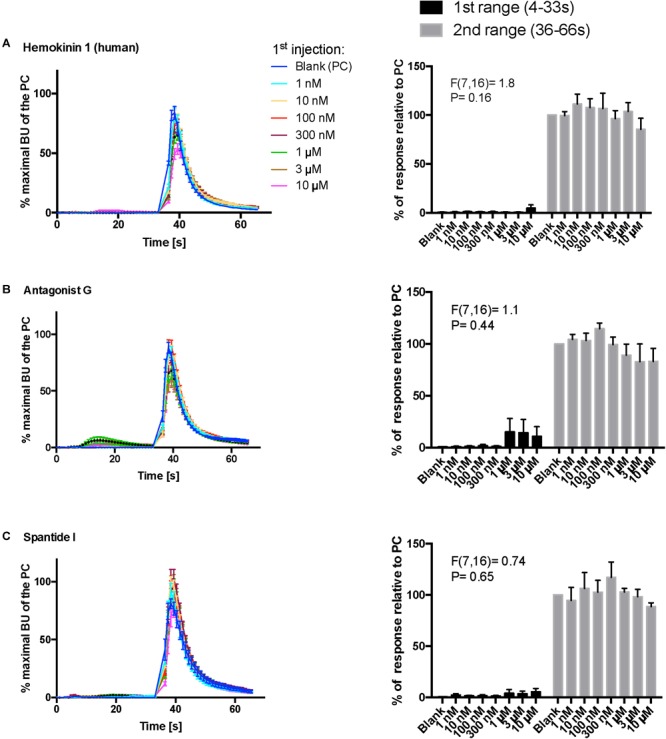
Kinetic dose-responses of the BMLK3 cell to three peptidomimetic ligands of mammalian neurokinin receptors in the “dual-addition” calcium bioluminescence assay. Kinetic bioluminescence responses of tick kinin receptor recombinant cells to **(A–C)** three peptidomimetics (1 nM–10 μM, *n* = 3). Two additions were performed by the two injectors built-in the Clariostar^TM^ plate reader (BMG LABTECH) as follows: the first addition of various concentrations of peptidomimetics (10×), or corresponding blank buffer, was performed at 2 s after initiation of the recording. The response to the first addition was recorded for 30 s with 1 s intervals (first range). At 32 s, the second addition of the agonist of tick kinin receptor (1 μM FFFSWGa) was performed in the same well, and the cellular response was recorded for another 30 s with 1 s intervals (second range). Curves on the left of each panel depict in different colors the kinetic response in bioluminescence units (BU) per second to each concentration (8–9 wells from three independent assays). The *Y*-axis represents the percentage BU of each concentration normalized to the maximal BU of the positive control (blank buffer + agonist, blue curve) (mean ± SEM). The histogram on the right of each panel shows sequentially the total BU responses of cells to the two additions, the first and second range recordings, respectively. The total responses in each range was expressed as the percentages of the average BU (per second) obtained in each of the two 30 s-ranges, relative to the average units to the PC in the second range (average BU of the blue curve at 35–65 s). In each independent assay, the total response to each concentration of each peptidomimetic in the first addition or to the agonist in the second addition was calculated as the average of 2–3 pseudo-replicate wells (*Y* axis: mean ± SEM, *n* = 3 independent assays). None of the tested compounds showed agonist activity (for **B** panel, first range, see explanation in text). Statistical differences in inhibitory activities between different concentrations of each compound were analyzed by one-way ANOVA (*n* = 3) but no statistical differences were detected.

### Phylogenetic Analysis of Kinin Precursors

To establish how conserved the kinin precursors are between ticks and other insect blood feeders, a phylogenetic Bayesian analysis was performed with the putative kinin precursors from eight tick species and those known from five blood-feeding insect species (Figure 4). Bhatt et al. (2014) validated the *in vivo* activity of the *R. prolixus* kinin sequences used to construct this tree, and the three aedeskinins are active on the cognate receptor (Pietrantonio et al., 2005). The kinins from *C. lectularius* were detected in the CNS by mass spectrometry by Predel et al. (2017).

The number of kinin paracopies in different arthropod species varied dramatically. However, the selection pressure behind these putative insertions or deletions is unclear. The number of kinin copies was high in ticks and some hematophagous insects, but not in all (Figure 4). With the support of the phylogenetic unrooted tree, we found that the kinin precursors from ticks and insects were separated into two different groups. Similarly, within each branch, arthropods that are phylogenetically closer to each other clustered in the same group, indicating substantial conservation of kinin precursors across broad taxonomic groups. The only exception was that a soft tick, *O. turicata*, clustered with *Ixodes* ticks (hard ticks) but with a posterior node probability of 0.76. Insect kinin precursors, even those from species with several paracopies as in the case of ticks (i.e., up to 15 in *R. prolixus*), were more similar among themselves than to tick kinin precursors (Supplementary Figure S4). Thus, the number of kinin paracopies, does not appear to differentiate sequences of ticks from those of insects.

### Small Molecule Screening on Recombinant Tick Kinin Receptor

Toward the discovery of novel “small-molecule” ligands (Lipinski, 2016) for the target GPCR, we initiated a small-scale screening with the dual-addition assay we developed. Because kinin receptors are invertebrate-specific GPCRs, there is no available commercial library of ligands from any ortholog mammalian GPCR (Figure 1). To explore the opportunity for discovery of antagonists, we selected a small-molecule library of known antagonists of those mammalian GPCRs that have the highest amino acid sequence identity to the tick kinin receptor. These are the neurokinin receptors (NK1-3 receptors), with approximately 36% amino acid sequence identity to the tick kinin receptor, as per Blast analyses in NCBI. The preliminary screening of fourteen small molecules was performed with an end-point calcium fluorescence assay. The screening of these small molecules was with 22 concentrations of antagonists prepared in 2% DMSO, performed on the BMLK3 cell line only. Four compounds, TKSM02, TKSM10, TKSM13 and TKSM14 showed some antagonistic activities, as they showed increasing values of fluorescence with decreasing concentrations (Supplementary Table S3, panel D, rows C, K, N, and O). It was noticed during the plate preparation that compounds TKSM04, TKSM05, TKSM08, TKSM09 and TKSM14 may have precipitated out when prepared in 2% DMSO at 1 mM. Indeed, fluorescence endpoint readings of some of them confirmed this suspicion due to randomness of their values. Therefore, a second small-molecule plate was made in 10% DMSO with ten selected compounds prepared in 10 serial concentrations (Supplementary Table S4, panel A). These small molecules included the four active small molecules from the first screen; those four more hydrophobic molecules that had precipitated; and the small molecules TKSM06 and TKSM07 that had showed erratic activity in the first screen (Supplementary Table S3, panel D, rows G and H). When TKSM02 and TKSM14 were tested after being prepared in 10% DMSO, they showed dose-dependent antagonistic activities that allowed the estimation of IC_50_s and confidence intervals (Figure 5A,D). TKSM02 acted as a full antagonist with IC_50_ = 45 μM (95% confidence interval of 31–61 μM) (Figure 5A). Three small molecules showed partial antagonism: TKSM14, with IC_50_ = 23 μM (95% confidence interval of 17–30 μM), only inhibited 60% of the agonist response at the highest concentrations tested of 35 and 50 μM (Figure 5D), and TKSM10 and TKSM13 did not have meaningful IC_50_ values because their 95% confidence intervals were too wide (Figure 5B,C). Nevertheless, TKSM10 exhibited antagonist activity at concentrations above 70 μM and inhibited 80% of agonist response when applied at 100 μM (Figure 5B). Although TKSM13 showed an overall weak antagonistic activity across concentrations, it acted as a potential antagonist (Figure 5C) as it inhibited approximately 50% of the agonist response at concentrations above 25 μM. The rest of the compounds re-tested (TKSM04-TKSM09) did not show any activity (Supplementary Table S4, panel D).

The small molecule library prepared for the second screening of potentially insoluble molecules (10% DMSO), was screened on both BMLK3 cells and “vector only” cells to test for the tick receptor specificity of the agonist responses observed after the first addition of small molecules, because the endogenous expression of NK receptors in CHO-K1 cells is assumed. Counter screening on vector only cells (Supplementary Table S4). confirmed that none of the agonistic responses observed after the first addition were specific for the tick kinin receptor because the vector only cells showed equivalent agonistic responses to these molecules as BMLK3 cells did (Supplementary Table S4, panel B, compare row F, wells 2-12 to row F, wells 22 to 13). Therefore, only antagonistic activities remain to be demonstrated in tick bioassays.

### Kinetic Responses to Selected Small Molecules and Peptidomimetics in a Dual-Addition Calcium Bioluminescence Assay

The primary screen using fluorescence provided only one end-point reading after 5 min of incubation with cells. A kinetic calcium bioluminescence assay was subsequently performed with molecules identified in the first screen. This was to determine the kinetic responses during the first 30 s after the addition of compound of unknown activity (small molecule or peptidomimetic) and the response after the subsequent addition of known agonist (1 μM FFFSWGa) (Figure 6). In contrast to the results of the fluorescence assay, nearly no agonist responses were detected in the kinetic assay in the first 30 s after the addition of small molecules (Figure 6A–E, right panel, black bars). The statistical analyses of antagonist activities based on the responses after the agonist addition were calculated by one-way ANOVA followed by Tukey’s multiple comparison test among all dosages. Consistent with the preliminary screen, TKSM02 was the most potent antagonist on the tick kinin receptor and inhibited 82.7 ± 1.8% of the FFFSWGa agonist response at 100 μM (Figure 6A; black curve in the left panel and in the histogram, last gray bar). The bioluminescence response of TKSM02 at 100 μM was significantly lower than the responses to the four lower concentrations tested from 100 nM–30 μM (histogram, four bars to the left), and significantly lower than the response to agonist only (Blank bar), as expected (*p* < 0.0001) (Figure 6A). A dose-dependent trend of antagonistic responses was observed for TKSM10 from 1 to 100 μM (Figure 6B, decreasing bar heights in histogram), with the lowest cell response being 84 ± 6% of the response to agonist only (16% inhibition). However, the increasing trend of inhibition with higher concentrations was not detected as statistically significant by ANOVA (*p* = 0.08). The ANOVA for TKSM13 detected significant variation in the response among concentrations (*p* = 0.02) and Tukey’s test detected a significant decrease in the response to the agonist of 72 ± 19% at 100 μM with respect to the 133 ± 17% response to 10 μM concentration (*p* < 0.05) (Figure 6C). Unexpectedly, TKSM14 did not show any antagonistic activity in the calcium bioluminescence assay, which was inconsistent with the result of the primary screen in the fluorescence assay. We then asked whether increasing the preincubation time to 5 min in the bioluminescence assay (previously, 30 s), similar to the fluorescence assay, could reveal TKSM14 antagonistic activity. Indeed, when the preincubation with TKSM14 was 5 min, a dose-dependent antagonistic response was detected after agonist addition (Figure 7A,B). Tukey’s test showed significant antagonistic activities of TKSM14 at both 50 μM (63 ± 8%) and 100 μM (55 ± 4%) which inhibited ∼ 40 and 45% of the agonist response (Figure 7A, orange and black curves on the left panel and last two bars on the histogram to the right). This activity matched the result observed in the first screen. This suggested that TKSM14 might have a lower binding affinity, reflected in the longer incubation time with the cells required to block the receptor.

Compound TKSM03 was chosen as a negative control for the bioluminescence assay with small molecules due to its lack of activity in the fluorescence screen. Its lack of activity was confirmed in the bioluminescence assay, as this compound did not decrease the agonist response even at the highest concentration tested of 100 μM (Figure 6E, histogram on right). The corresponding black curve on the left panel (Figure 6E) could be misleading at first sight, giving the impression that TKSM03 could be an antagonist, as the black curve appeared to be lower than other curves. However, it must be emphasized that although the maximal bioluminescence units per second at about 40 s is diminished with respect to the other concentrations, the area under the curve did not change with respect to that of the blank control (Figure 6E, right panel). This is why it is important to consider the total, integrated bioluminescence response and not only the peak response.

In addition, three peptidomimetic ligands of mammalian neurokinin receptors were tested using this approach. These ligands of NK receptors showed neither agonistic nor antagonistic activities on the tick kinin receptor within the tested concentration range (1 nM–10 μM) (Figure 8A–C) (ANOVA *p* > 0.1 for all three peptidomimetics). In Figure 8B, a slight agonistic activity of antagonist G at 1-, 3- and 10-μM can be detected, peaking at about 14 s; the same activity is reflected in the histogram to the right. However, this activity was only observed in the first independent replication and could not be seen in the two other independent replications. This agonist response was also not detected in the vector only cells (not shown). We do not know what could have caused this effect.

## Discussion

The insect kinins were named leucokinins after first being isolated from the roach *Leucophaea maderae* for their myotropic activity (Holman et al., 1986). The kinin neuropeptide signaling system is pleiotropic in insects, regulating physiological functions both at the central and peripheral levels. They regulate myotropic and diuretic activities, orchestrate innate behaviors during pre-ecdysis and influence feeding behavior (Veenstra et al., 1997; Kim et al., 2006; Kersch and Pietrantonio, 2011; Schooley et al., 2012; Kwon et al., 2016; Pietrantonio et al., 2018). However, the kinin functions in ticks have remained elusive. In the tick *R. microplus* LKR was immunolocalized on the midgut outer surface, and LKR-silenced females displayed variations in gut discoloration, had a reduction in body weight of ∼30%, reduced weight of their egg masses, and experienced decreased egg hatching. Thus, LKR silencing in female ticks caused a reproductive fitness cost, perhaps related to defects in heme metabolism because some guts from silenced females were completely white (Brock et al., 2019). In agreement, the LKR transcript of the tick *R. sanguineous* was apparently expressed at higher levels in the gut and salivary glands than in other tissues, as inferred from RT-PCR analyses (Lees et al., 2010).

Most information on tick GPCRs was obtained from recombinant receptor systems (Yang et al., 2013, 2015; Gross et al., 2015; Gondalia et al., 2016; Kim et al., 2018). Transcripts for tick GPCRs are often expressed *in vivo* at very low levels, which complicates their physiological characterization. For example, results of qRT-PCR after gene silencing is often highly variable in diverse tick tissues due to their normal low expression levels (Brock et al., 2019). To address this challenge, additional future approaches to elucidate the physiological function of the tick kinin system through loss-of-function experiments include the knockdown of the endogenous kinin neuropeptide transcript and a pharmacological approach to block receptor function. With these approaches in mind, we cloned the kinin cDNA from *R. microplus*, and developed a dual addition assay to discover antagonists of the receptor to block the response to endogenous ligands and impair the normal physiology of ticks (Figure 1). To expand our current knowledge on the kinin system of the cattle fever tick, *R. microplus*, we used a forward pharmacological approach (Figure 1). Herein, we integrated the cloning of the cDNA encoding the tick endogenous kinins from the synganglion of female *R. microplus* and predicted kinin sequences from other Acari.

Kinins arose before the shared common ancestor of Mollusca, Insecta and Acari, and the lymnokinin receptor (leucokinin-like) was functionally characterized in the pond snail, *Lymnaea stagnalis* (Cox et al., 1997; Grimmelikhuijzen and Hauser, 2012).

In the cattle fever tick, we identified a single cDNA that encodes 17 potential bioactive kinins, all different (Table 1). This is the first report for the cloning of a kinin-encoding cDNA from any tick species. Similar to *R. microplus*, the additional tick species analyzed also showed an expansion in the number of predicted kinins (Figure 3), underscoring the potential importance of the kinin system in ticks. Most of the *R. microplus* kinins featured the conserved C-terminal pentapeptide motif found in insects (FX_1_X_2_WGa). We predict the kinins reported in this study are active because they satisfy the minimal requirement for activity and we have experimental evidence of activity of similar kinin analogs and insect kinins on the *R. microplus* receptor expressed in CHO-K1 cells (Holmes et al., 2003; Taneja-Bageshwar et al., 2006, 2009; Xiong et al., 2019). However, the variable position X_1_ that in insect kinins is occupied by His, Asn, Ser or Tyr (Torfs et al., 1999), is different in some of the *R. microplus* kinins, featuring instead Gly, Ala, Thr, or Arg (Bold amino acids in Table 1). *R. microplus* retained the same variable residues as in the insect kinins in position X_2_ (Ser/Pro/Ala) (Torfs et al., 1999), with Pro being more frequent in tick kinins (Table 1). These seventeen predicted kinins await to be synthesized and tested in the receptor functional assay to verify their biological activities.

The genome analysis of *Ix. scapularis* identified a putative kinin-encoding gene and four genes encoding kinin receptors (Gulia-Nuss et al., 2016). In contrast, BLAST of *R. microplus* LKR against the updated genome assembly (Rm2.0; NCBI) of this species identified only one receptor and did not reveal kinin receptor paralogs. The same sequence of 1,481 bp was identified in two contigs (sequences ID LYUQ01138740.1 and LYUQ01085891.1), and it matched the 3′-half of the *R. microplus* LKR cDNA with 99.9% identity. Therefore, it appears that in *R. microplus* there is only one kinin receptor gene, or perhaps an identical duplicate. In the genomes of six non-tick chelicerate species (spiders, the house dust mite and a scorpion), five species have one gene for both the kinin peptide and receptor, except that the African social velvet spider (*Stegodyphus mimosarum*) has 3 LKR paralogs (Veenstra, 2016). The transcript expression level of kinin and its receptor(s) seems to be generally low in ticks because transcripts were not reported in transcriptomes from synganglia of females of *R. microplus* from Texas, *Ix. scapularis* and *O. turicata* (Egekwu et al., 2014, 2016; Guerrero et al., 2016). As the kinin receptor transcript level is low in ticks (as well as the level of receptor protein (Brock et al., 2019), it is tempting to speculate that kinin signaling may be regulated by changes in the expression and release of their expanded kinin peptide repertoire. A detailed temporal quantitative analysis of receptor and kinin precursor mRNA transcripts is still lacking. It is also yet unknown whether the high copy number of the tick kinins will result in increased peptide expression (i.e., concentration) or in sustained, constitutive expression. It is worth noting, however, that kinins are active at nanomolar levels (low concentrations) (Holmes et al., 2003; Xiong et al., 2019).

Kinins arose before the shared common ancestor of Mollusca, Insecta and Acari, and the lymnokinin receptor (leucokinin-like) was functionally characterized in the pond snail, *Lymnaea stagnalis* (Cox et al., 1997; Grimmelikhuijzen and Hauser, 2012). Among ticks, the Argasidae are considered a basal group to the Ixodidae (Geraci et al., 2007). Arthropod molecular phylogenetic studies have suggested Prostriata are more ancient than Metastriata (Jeyaprakash and Hoy, 2009). Although the pentapeptide core of tick kinins is largely conserved, it is noteworthy that more ancient species show higher conservation on the X_1_ position of this core, with asparagine (N), glycine (G) or serine (S) being more frequent. Further, although the Prostriata and Metastriata have a similar number of kinin paracopies (∼17), the tick kinins of the Metastriata showed more frequent amino acid substitutions than those of the Prostriata. Specifically, *Ix. scapularis* and *Ix. ricinus* (Prostriata) have multiple iterations of the same kinin sequence (DTFGPWGa) in the kinin precursor, whereas ticks from the Metastriata showed no repetition in the sequence of kinin paracopies (Table 1 and Figure 3A).

The unrooted phylogenetic tree revealed kinin sequence differences between ticks and insect blood feeders (Figure 4). Although the number of paracopies does not appear to differentiate kinin sequences of ticks from those of insects, we have experimental evidence that kinin receptors from insects and ticks have different ligand structure-activity relationships, as manifested in the differential potency of the same set of kinin analogs (Taneja-Bageshwar et al., 2006; Xiong et al., 2019). Therefore, differences in the sequences of the mature kinin peptides between ticks and insects further support this differential activation. Additionally, we reiterate that kinins have pleiotropic functions in insects and therefore, it is possible that they are similarly pleiotropic in ticks. We speculate this pleiotropism could be achieved in ticks either by diversification of receptors or ligands. In *Ixodes* (Prostriata), despite low variation in the kinin sequences, up to four kinin receptors may be present (Gulia-Nuss et al., 2016). In contrast, *Rhipicephalus microplus* appears to have only one receptor but exhibits high variation in the sequences of the kinin ligands (Table 1). As a mechanism for pleiotropism, there is evidence that in the mosquito renal organ different kinin analogs promote differential downstream tissue responses, either fluid secretion or changes membrane voltage (Schepel et al., 2010), apparently through the same receptor because only one kinin receptor has been identified in this tissue (Lu et al., 2011a).

To advance the discovery of novel ligands toward application, we tested potential synthetic ligands (small molecules and peptidomimetics) of mammalian neurokinin receptors on the recombinant tick receptor (Figure 1). Our previous success with a kinin mimetic, named 1728, that elicits aversive behavior in the mosquito and inhibits the sugar neuron provided the proof of principle to pursue this approach (Kwon et al., 2016). The NK receptors are considered “pseudo-orthologs” of the arthropod kinin receptor because while tachykinins exist in both mammals and arthropods, the kinin system is arthropod-specific (Nachman et al., 1999). To accelerate discovery and reduce the cost of screening a commercial GPCR-specific small molecule library and selected peptidomimetics, we developed a dual-addition functional assay using two reporters for calcium, in fluorescence and bioluminescence modes, respectively. A comparable pipeline was developed by Ejendal et al. (2012) for validating the dopamine receptor of *Ix. scapularis* as acaricide target, except their functional assay detects cAMP as the secondary messenger. Using a reverse pharmacological approach, Duvall et al. (2019) recently discovered a novel small molecule ligand of the mosquito neuropeptide receptor, NPY receptor, which discourages the biting behavior of mosquito (Duvall et al., 2019).

In this study, through the primary screening of a small molecule library of NK receptor antagonists, and their secondary validation by a bioluminescence assay, a small molecule, named TKSM02, was identified as a “full antagonist” of the tick kinin receptor with an IC_50_ of 45 μM (Figure 5A), as it fully blocked receptor activity at 100 μM. Although of weak potency, we believe this is the first antagonist reported for a kinin receptor of any arthropod species. However, it should be noted that we used a generic kinin receptor agonist, FFFSWGa, in the antagonist screen. Therefore, the antagonists identified with this screen may not similarly antagonize endogenous tick kinins on the cognate receptor. Overall, ligands of NK receptors displayed more than a 10,000-fold reduced potency on the tick kinin receptor. The quantitative functional data reported here will provide valuable information to constrain the ligand-receptor interaction surface in computational modeling.

Agonists and antagonist peptidomimetics of NK receptors were tested: hemokinin 1 (human) is an agonist of the NK1 receptor with active concentration at the nanomolar level, antagonist G is a broad antagonist on NK receptors, and spantide I is a selective potent antagonist of the NK1 receptor (Supplementary Table S2). Our results provided additional functional evidence that the tick kinin receptor is pharmacologically different from the insect tachykinin receptor as none of the peptidomimetic ligands of NK receptors showed any activity on the tick kinin receptor. However, spantide I antagonizes insect tachykinins in recombinant tachykinin receptors of the stable fly and fruit fly at 1 and 50 μM, respectively (Torfs et al., 2002; Poels et al., 2009).

In summary, we report for the first time putative tick kinin sequences. Further, our small molecule screening results confirmed in one direction the target selectivity of the kinin receptor for arthropod vector control, as only one of the small molecule NK antagonists was active. The dual addition assay we developed is amenable and ready for the high throughput screening of random small molecule libraries on the tick kinin receptor or other tick neuropeptide receptors signaling through the calcium cascade. While receptor “hits” of random molecules are expected, the next frontier is to develop suitable tick bioassays to elucidate the physiological functions of kinins that could be impaired by these synthetic ligands.

## Data Availability

The DNA sequence of the PCR product verifying the *R. microplus* kinin gene sequence with mRNA sequence of the kinin precursor were uploaded in NCBI with GenBank accession number (MK875970). The raw data of the fluorescence dual-addition assay are under Supplementary Tables S3, S4. The raw data of the calcium bioluminescence assay can be made available upon request.

## Author Contributions

PP and CX designed the study, developed the fluorescence assay, performed the data analyses and wrote the manuscript. CX was responsible for performing the molecular cloning, cell culture and calcium bioluminescence assay. DB advised on composition of small molecule library. CX and DB performed the 384-well-plate calcium fluorescence assay. All authors edited and approved the final manuscript.

## Conflict of Interest Statement

The authors declare that the research was conducted in the absence of any commercial or financial relationships that could be construed as a potential conflict of interest.

## Footnotes

1 https://www.ncbi.nlm.nih.gov/

2 http://www.cbs.dtu.dk/services/SignalP/

3 https://weblogo.berkeley.edu/logo.cgi

4 https://web.expasy.org/translate

5 http://www.neurostresspep.eu/home

6 https://mafft.cbrc.jp/alignment/server/
