# Supplementary material for: The Cattle Fever Tick, Rhipicephalus microplus, as a Model for Forward Pharmacology to Elucidate Kinin GPCR Function in the Acari
Source: Front Physiol. 2019 Aug 7;10:1008. doi: 10.3389/fphys.2019.01008 (PMC6692460; doi:10.3389/fphys.2019.01008)
Supplement: Supplementary file 2 [file Image_1.pdf]

Title: The cattle fever tick, *Rhipicephalus microplus*, as a model for forward pharmacology to elucidate kinin GPCR function in the Acari

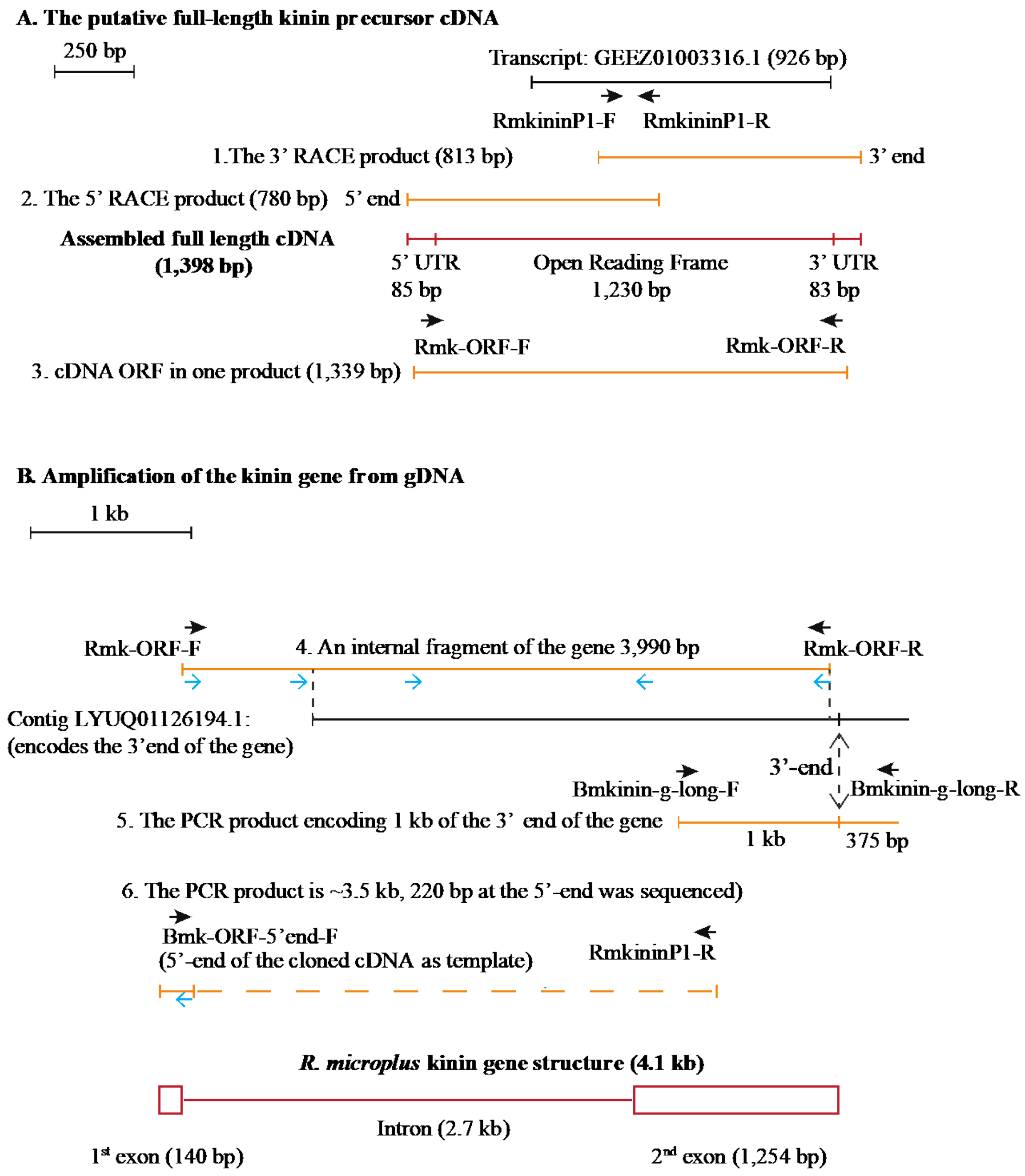

Supplementary Figure 1. The workflow for cloning the putative kinin precursor cDNA from *Rhipicephalus microplus* and verification of the gene structure by sequencing genomic PCR products.

In this figure, within the panels A and B, all lines representing sequences are aligned vertically to the corresponding sequence location. Notice a different scale in bp on the left of each panel.

The solid black lines represent either transcriptome (A) or genomic (B) sequences identified through local NCBI BLAST using the putative kinin precursor (amino acid sequence) from *Ixodes scapularis* as query. The black arrows denote the direction and relative location of the primers on the template above the primers. The solid orange lines depict the sequenced PCR products obtained in this study. Red lines and boxes represent the deduced full-length cDNA (A) or the kinin gene (B) obtained by assembling the sequenced PCR products.

The blue arrows denote the position of primers used for sequencing. The dashed orange line depicts the region of a PCR product which was not sequenced (sequencing was not necessary because the overlapping sequence of ~200 bp at the 5' end was enough to align this product with the PCR product in reaction 4 above). **(A) The putative full-length kinin precursor cDNA from *R. microplus***: Cloning of 5'- and 3'- RACE PCR products. 1-2). A transcript predicted to encode a partial sequence of the putative kinin precursor was used to design a pair of gene-specific primers for the 3'- and 5'- RACE PCRs. The sequences of these 3'- and 5'- RACE PCR products (1-2) were aligned with DNASTAR (Lasergene, Madision, WI) to assemble the full-length cDNA. (3). A pair of primers was designed outside of the open reading frame (ORF) and were used to amplify the cDNA ORF in one product. **(B) Amplification of the kinin gene from genomic DNA.** (4). First, we amplified an internal fragment of the gene with a pair of gene-specific primers. (5). Secondly, the 3'-end of the kinin gene was amplified with a gene-specific forward primer and a reverse primer located outside of the ORF 3'-end. The sequence of this latter reverse primer was obtained from a genomic contig encoding the 3'-end fragment of the kinin gene (black line). (6). The 5'-end of the kinin gene was amplified using a forward primer which binds to the 5'-end of the cDNA, and with a reverse primer within the ORF. The DNA sequences from the PCR products above (4-6) were aligned with DNASTAR to obtain the sequence of the kinin gene shown in red: boxes represent exons, and the solid line represents the intronic region. The exons were identified by aligning the cDNA sequence with the gene sequence.

```
5'  ACATGGGGaGCAACAGGCGCTGCTAGAGGCCCCATCGACGGCAGCGCGAAGCGTTGGCATCGCCATCGTGTCCAGCACAG   80
5'  CAGCCATGGGAACGGTACTGCACCGTCGCGGGGCTGCTGGTGCTAACTTCACCGTCGGGGAATCACTTGTGCTGCTTCTT   160
    M G T V L H R R G A A G A N F T V G E S L V L L L
    1 2 3 4 5 6 7 8 9 10 11 12 13 14 15 16 17 18 19 20 21 22 23 24 25

5'  GTCCTGGGGGTCATTGCGTTCCCATTAGACGCCTGGGCTCAGCACGTCGTGGGCAGCGACGAGGCCCGCTCCCTACTTTC   240
    V L G V I A F P L D A W A Q H V V G S D E A R S L L S
    26 27 28 29 30 31 32 33 34 35 36 37 38 39 40 41 42 43 44 45 46 47 48 49 50 51 52

5'  AAGAGGTGGTGACACGCTAATCCGCTGGAACATCTCGCCCGCCGCGCTGCAGCACATGCGTTCCGAAAGCTTCAAGCGCC   320
    R G G D T L I R W N I S P A A L Q H M R S E S F K R
    53 54 55 56 57 58 59 60 61 62 63 64 65 66 67 68 69 70 71 72 73 74 75 76 77 78

5'  AGTTCAGTCCATGGGGCGGCAAGCGGAACGCCGCCGCATTCGAATCCCTGCTGGACGCAGCCGGCAATCAGGAGAGCCAC   400
    Q F S P W G G K R N A A A F E S L L D A A G N Q E S H
    79 80 81 82 83 84 85 86 87 88 89 90 91 92 93 94 95 96 97 98 99 100 101 102 103 104 105

5'  CGGCACCGTCTGGCAGCGGATGCCTCGTACAAGGTCCGTCGTTTGCACCCTGTGGACATCGCAGTGAGGGCCGCCGATTT   480
    R H R L A A D A S Y K V R R L H P V D I A V R A A D L
    106 107 108 109 110 111 112 113 114 115 116 117 118 119 120 121 122 123 124 125 126 127 128 129 130 131 132

5'  GTTCAGTCCATGGGGCGGCAAAAGAACGGACGACAAGAAGGACAAAGACCAGACGTTCAACCCTTGGGGAGGCAAGAGGG   560
    F S P W G G K R T D D K K D K D Q T F N P W G G K R
    133 134 135 136 137 138 139 140 141 142 143 144 145 146 147 148 149 150 151 152 153 154 155 156 157 158

5'  CAGGAGACCACTTCGGTTCCTGGGGAGGCAAGAGGGACACGTTCAGCGCGTGGGGAGGCAAGCGGCAGCAGGACAGCAAG   640
    A G D H F G S W G G K R D T F S A W G G K R Q Q D S K
    159 160 161 162 163 164 165 166 167 168 169 170 171 172 173 174 175 176 177 178 179 180 181 182 183 184 185

5'  AACGCCTTCAGTCCGTGGGGAGGYAAGAGAGCCGTCAGAAGCCCGACGGCCAGAAACGACGCCGCTAGGGCCAAGCAGGA   720
    N A F S P W G G K R A V R S P T A R N D A A R A K Q E
    186 187 188 189 190 191 192 193 194 195 196 197 198 199 200 201 202 203 204 205 206 207 208 209 210 211 212

5'  GGACGGAGAGGAGGACGAAGAGCGGAGCTTCGCACCTTGGGGAGGAAAGAGGGGAACCGGGGAAGACCAGGCGTTTTCTC   800
    D G E E D E E R S F A P W G G K R G T G E D Q A F S
    213 214 215 216 217 218 219 220 221 222 223 224 225 226 227 228 229 230 231 232 233 234 235 236 237 238

5'  CATGGGGAGGAAAAAGGGGCGACGACGGGGATACGTCTTTCACGCCCTGGGGAGGCAAGCGCGATGACCGCTTCAATCCA   880
    P W G G K R G D D G D T S F T P W G G K R D D R F N P
    239 240 241 242 243 244 245 246 247 248 249 250 251 252 253 254 255 256 257 258 259 260 261 262 263 264 265

5'  TGGGGAGGCAAGCGAGAAGGACCCTTCAGCCCCTGGGGAGGTAAGAGGGATGGCTCGAACAAGGAAGGATTCTTCAACCC   960
    W G G K R E G P F S P W G G K R D G S N K E G F F N P
    266 267 268 269 270 271 272 273 274 275 276 277 278 279 280 281 282 283 284 285 286 287 288 289 290 291 292

5'  TTGGGGAGGCAAGCGCGGAGCAGACGACCCGTTCAACCCTTGGGGAGGCAAGAGGCAGGATTCGTTCAATCCTTGGGGAG   1040
    W G G K R G A D D P F N P W G G K R Q D S F N P W G
    293 294 295 296 297 298 299 300 301 302 303 304 305 306 307 308 309 310 311 312 313 314 315 316 317 318

5'  GCAAGAAGGAAGATGGAGTCTTCAGGCCTTGGGGTGGAAAGAAGGAAGACAACGTCTTTAGGCCTTGGGGAGGCAAAAAG   1120
    G K K E D G V F R P W G G K K E D N V F R P W G G K K
    319 320 321 322 323 324 325 326 327 328 329 330 331 332 333 334 335 336 337 338 339 340 341 342 343 344 345

5'  GAGGGCAACGTGTTTGGGCCTTGGGGAGGCAAAAGAGAGGACGCGACACCGAGCCTTTCCGTGAGTAGGGCATTAAACTA   1200
    E G N V F G P W G G K R E D A T P S L S V S R A L N Y
    346 347 348 349 350 351 352 353 354 355 356 357 358 359 360 361 362 363 364 365 366 367 368 369 370 371 372

5'  CGGTGGTCCAGCAAGCCATGACGTGGATGCAGGGTCCTTGCGCAAGAAGCGAGATTCTTCTGCGAGCGAACAAAAGGGTA   1280
    G G P A S H D V D A G S L R K K R D S S A S E Q K G
    373 374 375 376 377 378 379 380 381 382 383 384 385 386 387 388 389 390 391 392 393 394 395 396 397 398

5'  CGACGTCGAACGGTGAGAAAAGTGGGAGGACGTAACCACTGACGCGAGTCACTGTAGCGAAGCGCCCGTTCGAAAAACAT   1360
    T T S N G E K S G R T .
    399 400 401 402 403 404 405 406 407 408 409 410

5'  CTCATCGCACGTCCCAAAAAAAAAAAAAAAAAAAAAAA                                             1398
```

Supplementary Figure 2. Cloned cDNA sequence and predicted amino acid sequence of putative kinin precursor from *Rhipicephalus (Boophilus) microplus.*

The full-length cDNA sequence is 1,398 bp, with 1,227 bp ORF that encodes a 409 amino acid residue protein. The predicted enzyme cleavage sites are boxed. Seventeen kinin sequences were predicted. See Fig. 3.

```
Majority           ----LRXR-AXX---------XXXLXX----MSXXXLXQXVXXSDGDXXXXSRXGDXXIRWNISPATLQHMRS--ESFKR
                           10        20        30        40        50        60        70        80
O.turicata         --MAPAARSSQT---------FCTLRYLILSISLLASYASVEESDGDSRDDDSFEDMVIRWNISPDTLQRLRARREAIAR 69
H.dromedarii       ------------------------------------------------------------------------MRS--ESFKR 8
D.variabilis       ------------------------------------------------------------------------MRS--ESFKR 8
A.sculptum         MG--LRRRAAAG------ETLLLRVFLVASVLSLDVSAQHMVGSDEARSLLSRGGDTLIRWNISPATLQHMRS--ESFKR 70
R.sanguineus       MGTVLRRRGAAGANFAVGETLMLQLLLVVIAFSSDAWAQHVVGSDEARSLLSRGGDTLIRWNISPAALQHMRS--ENFKR 78
R.microplus        MGTVLHRRGAAGANFTVGESLVLLLVLGVIAFPLDAWAQHVVGSDEARSLLSRGGDTLIRWNISPAALQHMRS--ESFKR 78
Ix.ricinus         --MELRGHDAQELR------FLWTLLATTFLMSMGGLCQDVDSGSGDLGRSSRVGESFIRWNISPATLQHMRS--ESFKR 70
Ix.scapularis-mRNA --MELRGHDAQELR------FLWTLLATTFLMSMGGLCQDVDSGSGDLGRSSRVGESFIRWNISPATLQHMRS--ESFKR 70
Ix.scapularis-gDNA -------------------------------MSMGGLCQDVDSGSGDLGRSSRVGESFIRWNISPATLQHMRS--ESFKR 47

Majority           QFSP-WGGKRN-AAXDSLLDXAGXQXSXRHLLAADASYKVRRLHXGD-QPRD-DSFNPWGGKRXXDK-------------
                           90       100       110       120       130       140       150       160
O.turicata         PFVPRWGNQIQG---DAAVGKPYGN------------YRDVWIYGK----KD---------------------------- 102
H.dromedarii       QFSP-WGGKRNAALLESLLDSPGNQESRRHHLAADASYKVRRLHPA--GVRDADSFNPWGGKRTDDKKDKDQTFNPWGGK 85
D.variabilis       QFSP-WGGKRNTAAFESLLDSPGDQQSHRHQLAADASYKVRRLHHTG-SPRNADSFS------------------P---- 64
A.sculptum         QFSP-WGGKRN-AAFDYLLDAPGGD--H-QVADAASSYKVRRVHHTP-SLRN---------------------------- 116
R.sanguineus       QFSP-WGGKRNTAAFESLLDSAGNQESRRHHLAADASYKVRRLHPASIGVRDADSFSPWGGKRTDDKKDKDQTFNPWGGK 157
R.microplus        QFSP-WGGKRNAAAFESLLDAAGNQESHRHRLAADASYKVRRLHPVDIAVRAADLFSPWGGKRTDDKKDKDQTFNPWGGK 157
Ix.ricinus         QFSP-WGGKRG--VLDQALPTAHRLSGPLYLYKALHSPGAMGERRGDKQPDD-ETFNPWGGKRENDK------------- 133
Ix.scapularis-mRNA QFSP-WGGKRG--VLDQALPTAHRLSGPLYLYKALHSPRAMGERRGDKQPDD-EAFNPWGGKRENDK------------- 133
Ix.scapularis-gDNA QFSP-WGGKRG--VLDQALPTAHRLSGPLYLYKALHSPRAMGERRGDKQPED-EAFNPWGGKRENDK------------- 110

Majority           --------------------------------------------------------------DXERSFAPWGGKRG----
                          170       180       190       200       210       220       230       240
O.turicata         -----------------------------------------------------------------AHFNPWGGKRDGTHT 117
H.dromedarii       R-------------------------------------------------DSVRVKQEDGEEDEERSFAPWGGKRG---- 112
D.variabilis       ----------------------------------------------------------------ERSFAPWGGKRG---- 76
A.sculptum         ------------------------------------------------------------------VFAPWGGKR----- 125
R.sanguineus       RAAAGDRFGSWGGKRETFNAWGGKRQQD-KNSFSPWGGKRAVRSPTARNEDAARARQDDGEEDEERSFAPWGGKRG---- 232
R.microplus        R--AGDHFGSWGGKRDTFSAWGGKRQQDSKNAFSPWGGKRAVRSPTARN-DAARAKQEDGEEDEERSFAPWGGKRG---- 230
Ix.ricinus         --------------------------------------------------------------DKELSFNPWGGKRGTFSS 151
Ix.scapularis-mRNA --------------------------------------------------------------DKELSFNPWGGKRGSFSS 151
Ix.scapularis-gDNA --------------------------------------------------------------DKELSFNPWGGKRGSFSS 128

Majority           -XGEDXAFS------------------------------PWGGKRXDDGD-----TXFXPWGGKREDR---FNPWGGKRE
                          250       260       270       280       290       300       310       320
O.turicata         RSGLDDSSG------------------------------SWSEKK----D------NFNPWGGKKEAS---FNPWGGKRD 154
H.dromedarii       -AGEDQAFS------------------------------PWGGKKRDDGD-----TSFAPWGGKREDR---FNPWGGKRE 153
D.variabilis       -AAEDQAFS------------------------------PWGGKRDSEED-----TSFAPLGGKREDR---FNPWGGKRE 117
A.sculptum         -AAEDRAFS------------------------------PWGGKRGGDGDGG---ETFAPWGGKREDR---FNPWGGKRE 168
R.sanguineus       -TGEDQAFS------------------------------PWGGKRGDDGD-----TSFTPWGGKREDR---FNPWGGKRE 273
R.microplus        -TGEDQAFS------------------------------PWGGKRGDDGD-----TSFTPWGGKRDDR---FNPWGGKRE 271
Ix.ricinus         WGGKRDTFG------------------------------PWGGKRDTFGAWGGKRDTFGPWGGKRQDKESGFNPWGGKRE 201
Ix.scapularis-mRNA WGGKRDTFG------------------------------PWGG----------KRDTFGPWGGKRQDKESGFNPWGGKRE 191
Ix.scapularis-gDNA WGGKRDTFGSWGGKRDTFGPWGGKRDTFGPWGGKRDTFGPWGGKRDTFGPWGGKRDTFGPWGGKRQDKESGFNPWGGKRE 208

Majority           GPFNPWGGKRE--------GSNKEGFFNPWGGKRGADDS-FNPWGGKR-------EDSFNPWGGKXX--XDTAFXPWGGK
                          330       340       350       360       370       380       390       400
O.turicata         N-FNPWGGKKD--------------NFNPWGGKK--DSSIFNPWGGKRSDGSKDKELSFNPWGGKRQDGVGTIFGPWGGK 217
H.dromedarii       GPFNPWGGKRD--------GSNKEGFFNPWGGKRGADDS-FNPWGGKRG-----ADDSFNPWGGKR---QD-SFNPWGGK 215
D.variabilis       GPFNPWGGKRE--------GSNKEGFFNPWGGKRGADDS-FNPWGGKR-------QDSFNPWGGKR---ESGVFRPWGGK 178
A.sculptum         GPFNPWGGKRE--------GSNKEGFFNPWGGKRGADDS-FNPWGGKRG-----ADDAFNPWGGKGG--ADAAFNPWGGK 232
R.sanguineus       GPFNPWGGKRD--------GSNKEGFFNPWGGKRGADDS-FNPWGGKR-------QDSFNPWGGKR---EDGVFRPWGGK 334
R.microplus        GPFSPWGGKRD--------GSNKEGFFNPWGGKRGADDP-FNPWGGKR-------QDSFNPWGGKK---EDGVFRPWGGK 332
Ix.ricinus         DPFNPWGGKKEDKNAFSPWGGKREQNFNPWGGKKTSKDSTFSPWGGKR-------EGPFNPWGGKKGD-SDTAFAPWGGK 273
Ix.scapularis-mRNA DPFNPWGGKKEDKNAFSPWGGKREQNFNPWGGKKTTKDSTFSPWGGKR-------EGPFNPWGGKKGD-SDTAFAPWGGK 263
Ix.scapularis-gDNA DPFNPWGGKKEDKNAFSPWGGKRDQNFNPWGGKKTTKDSTFSPWGGKR-------EGPFNPWGGKKGD-SDTAFAPWGGK 280
```

```
Majority            RED-VFNPWGGKRD----DXVFSPWGGKRED-------------FGXXA---DXLEDAXSXRKKRDSSXSEHKGTTSXG

                             410       420       430       440       450       460       470       480

O.turicata          REN-LFNPWGGKREKS--EMTFNPWGGKKSE------------LAFSPWM--------SASSRIKRDSTINTDMFIQHPK 274
H.dromedarii        REDGVFRPWGGKKD----DNVFRPWGGKKED----------NV--FGPWG---GKREDAGSLRKKRDSSTSEHKVTTSHG 276
D.variabilis        KEDKVFGPWGGKRD----DDVFGPWGGKREQVSSSSSHSAGRGFPFGGAT---EHGVDAESLRKKRDSSMSEHQVTTSYS 251
A.sculptum          RQD-SFNPWGGKRE----DVVFRPWGGKKED----N--------VFRPWG---GKKEDN-VFRPWGGKRQGD--GFKPWG 289
R.sanguineus        KEDNVFRPWGGKKE----DNVFAPWGGKREDALPS--LYVGRGFNYGGTA---SHGVDAGSLRKKRDSSTSEHKGTTSYG 405
R.microplus         KEDNVFRPWGGKKE----GNVFGPWGGKREDATPS--LSVSRALNYGGPA---SHDVDAGSLRKKRDSSASEQKGTTSNG 403
Ix.ricinus          RDN-NFNPWGGKRDNGNKDSSFSPWGGKRES-------------FGVQASDPDSLEDHSPSRNKRDSSSRVPKTKNSAR 338
Ix.scapularis-mRNA  RDN-NFNPWGGKRDNGNKDSSFSPWGGKRES-------------FGVQALDPDSLEDHSPSRNKRDSSSRVPKTKNSAQ 328
Ix.scapularis-gDNA  RDN-NFNPWGGKRDNGNKDSSFSPWGGKRES-------------FGVQALDPDSLEDHSPSRNKRD               332

Majority            GKSGST----

                             490

O.turicata          VQKASPRPL                                                                         283
H.dromedarii        GKSASA                                                                            282
D.variabilis        GKSGGT                                                                            257
A.sculptum          GKREEDT                                                                           296
R.sanguineus        GKSGST                                                                            411
R.microplus         EKSGRT                                                                            409
Ix.ricinus          SAISSVAKTF                                                                        348
Ix.scapularis-mRNA  STIRSVAKTF                                                                        338
Ix.scapularis-gDNA                                                                                    332
```

Supplementary Figure 3. Multiple amino acid sequence alignment of the deduced kinin precursors from eight tick species using Clustal W method by MegAlign (Lasergene, Madision, WI).

There are two sequences from *Ixodes scapularis,* the first one was deduced from three transcriptome sequences (GBBN01023680.1, GGIX01408871.1, GBBN01027580.1), and the second was deduced from a genome scaffold (DS680282) (Gulia-Nuss and Caffrey, 2016). The amino acid sequence from the latter lacks 23 residues at the N- terminus and also 23 residues at the C-terminus. The sequence deduced from the transcriptome (*Ix. scapularis*-mRNA) misses some internal amino acids within positions 250-293.

Gulia-Nuss, M., Nuss, A.B., Meyer, J.M., Sonenshine, D.E., Roe, R.M., Waterhouse, R.M., Sattelle, D.B., de la Fuente, J., Ribeiro, J.M., Megy, K., Thimmapuram, J., Miller, J.R., Walenz, B.P., Koren, S., Hostetler, J.B., Thiagarajan, M., Joardar, V.S., Hannick, L.I., Bidwell, S., Hammond, M.P., Young, S., Zeng, Q., Abrudan, J.L., Almeida, F.C., Ayllon, N., Bhide, K., Bissinger, B.W., Bonzon-Kulichenko, E., Buckingham, S.D.,, and Caffrey, D.R., Caimano, M.J., Croset, V., Driscoll, T., Gilbert, D., Gillespie, J.J., Giraldo-Calderon, G.I., Grabowski, J.M., Jiang, D., Khalil, S.M., Kim, D., Kocan, K. M., Koci, J., Kuhn, R.J., Kurtti, T.J., Lees, K., Lang, E.G., Kennedy, R.C., Kwon, H., Perera, R., Qi, Y., Radolf, J.D., Sakamoto, J.M., Sanchez-Gracia, A., Severo, M.S., Silverman, N., Simo, L., Tojo, M., Tornador, C., Van Zee, J.P., Vazquez, J., Vieira, F.G., Villar, M., Wespiser, A.R., Yang, Y., Zhu, J., Arensburger, P., Pietrantonio, P.V., Barker, S.C., Shao, R., Zdobnov, E.M., Hauser, F., Grimmelikhuijzen, C.J., Park, Y., Rozas, J., Benton, R., Pedra, J.H., Nelson, D.R., Unger, M.F., Tubio, J.M., Tu, Z., Robertson, H.M., Shumway, M., Sutton, G., Wortman, J.R., Lawson, D., Wikel, S.K., Nene, V.M., Fraser, C.M., Collins, F.H., Birren, B., Nelson, K.E., Caler, E., Hill, C.A. (2016). Genomic insights into the *Ixodes scapularis* tick vector of Lyme disease. *Nat. Commun.* 7**,** 10507.

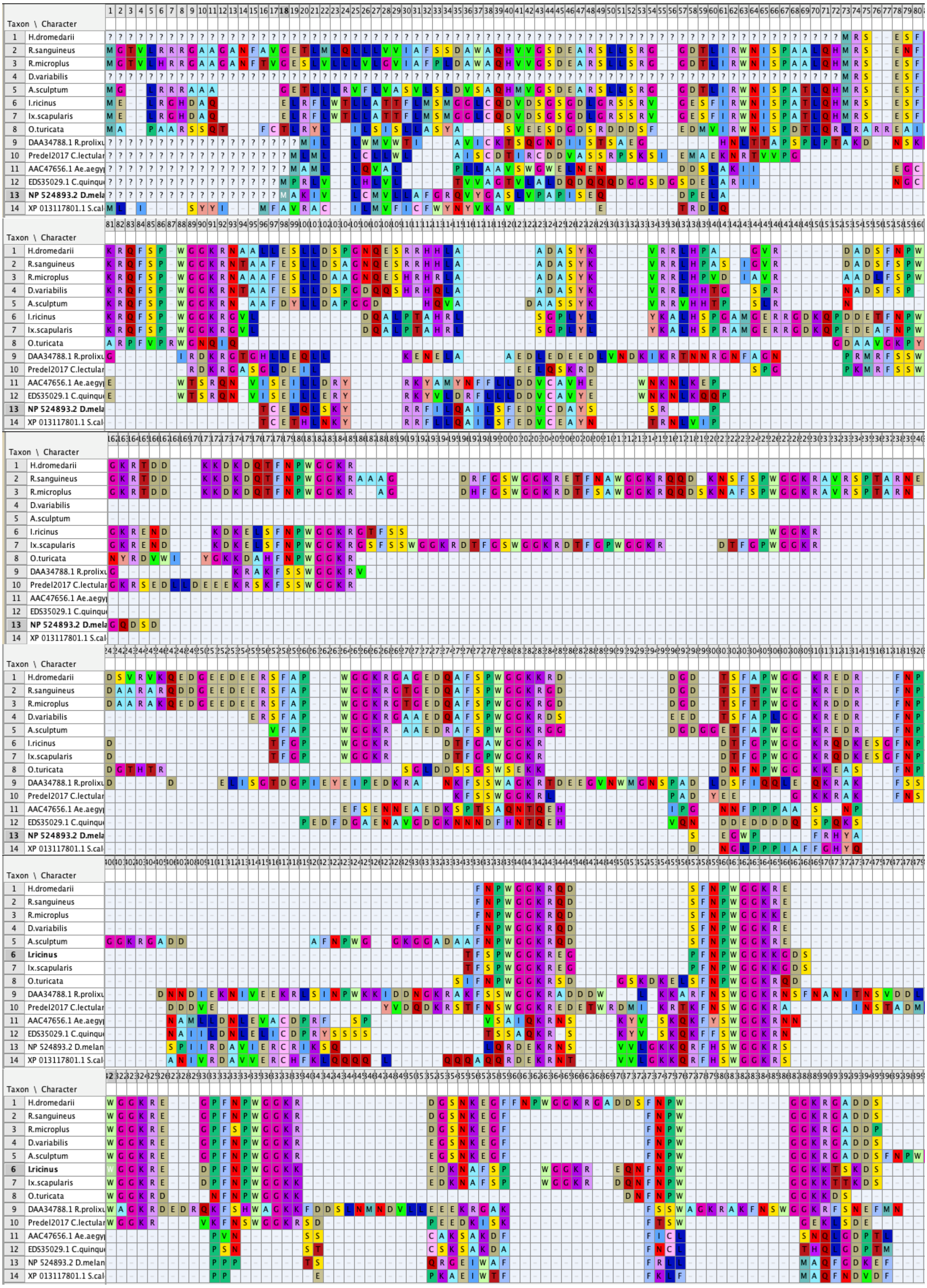
Taxon \ Character
H.dromedarii
R.sanguineus
R.microplus
D.variabilis
A.sculptum
I.ricinus
Ix.scapularis
O.turicata
DAA34788.1 R.prolixus
Predel2017 C.lectularius
AAC47656.1 Ae.aegypti
EDS35029.1 C.quinquefasciatus
NP 524893.2 D.melanogaster
XP 013117801.1 S.calcitrans

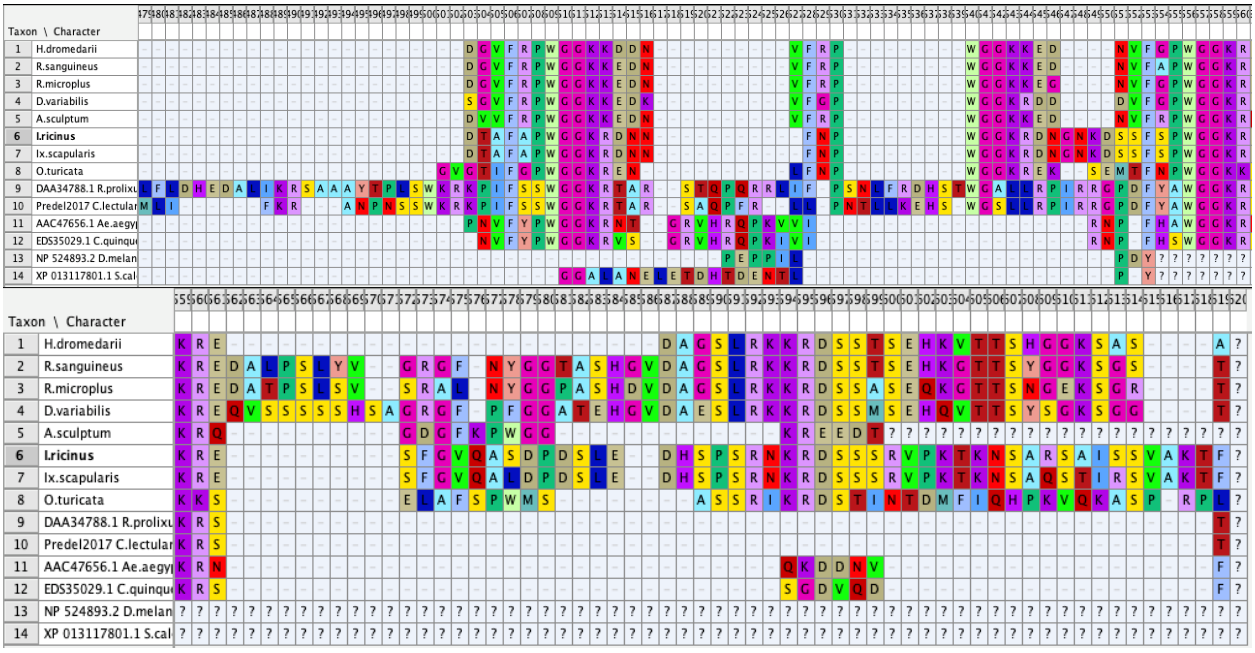

Supplementary Figure 4. Above is the alignment of the fourteen kinin precursors that were used for the phylogenetic analysis, as shown in the Mesquite window (Version 3.6).
